# TRPs—NCX2 Axis Jointly Preserves Pancreatic Beta Cell Susceptibility/Sensitivity to Elevated Glucose

**DOI:** 10.3390/cells15191766

**Published:** 2026-09-28

**Authors:** Adlet Sagintayev, Furkan E. Oflaz, Andra Kanlinuro, Savina van Amsterdam, Benjamin Gottschalk, Rene Rost, Zhanat Koshenov, Roland Malli, Wolfgang F. Graier

**Affiliations:** 1 Gottfried Schatz Research Center, Molecular Biology and Biochemistry, Medical University of Graz, Neue Stiftingtalstrasse 6/4east, 8010 Graz, Austria; adlet.sagintayev@medunigraz.at (A.S.); furkan.oflaz@medunigraz.at (F.E.O.); andra.kanlinuro@medunigraz.at (A.K.); savina.van-amsterdam@medunigraz.at (S.v.A.); benjamin.gottschalk@medunigraz.at (B.G.); rene.rost@medunigraz.at (R.R.); zhanat.koshenov@medunigraz.at (Z.K.); 2 Core Facility Bioimaging, Center for Medical Research (ZMF), Medical University of Graz, 8010 Graz, Austria; roland.malli@medunigraz.at; 3 BioTechMed-Graz, 8010 Graz, Austria

**Keywords:** beta cell, ER Ca^2+^ leak, NCX, TRPC3, TRPV4, ER Ca^2+^ refilling, glucose-stimulated insulin secretion

## Abstract

*Background:* For appropriate glucose sensing, pancreatic beta-cells essentially establish a constant mitochondria-directed endoplasmic reticulum (ER) Ca^2+^ leak via phosphorylated presenilin-1 (PS1). In the current work, we investigated how cells maintain the ER Ca^2+^ content during this continuous Ca^2+^ leak. *Methods:* Ca^2+^ imaging for ER, cytosolic and subplasmalemmal Ca^2+^, high resolution respirometry, and ELISA for insulin secretion were used. *Results:* A novel ER Ca^2+^ refilling mechanism driven by reverse-mode NCX2, which is fueled by local Na^+^ influxes through TRPC3 and TRPV4 channels that are independent of STIM-ORAI1 was found in pancreatic rodent and human beta cell lines. Disrupting this TRPs-NCX2 axis reduces subplasmalemmal Ca^2+^ levels, abolishes glucose-induced cytosolic Ca^2+^ oscillations, suppresses glucose-triggered elevation in mitochondrial energetics, and impairs first-phase insulin secretion. *Conclusions:* We identified a novel, ORAI1-independent “TRPs-NCX2” relay that couples Na^+^ influx via TRPC3 and TRPV4 to NCX2 reverse mode to maintain ER Ca^2+^ stores during physiological basal ER Ca^2+^ leakage, which is fundamental to beta-cell responsiveness to elevated glucose.

## 1. Introduction

The ability of pancreatic β-cells to sense changes in blood glucose concentration and secrete insulin accordingly relies on a finely tuned interplay between cellular metabolism and Ca^2+^ signaling. Upon glucose uptake and metabolism, the rise in intracellular ATP leads to closure of ATP-sensitive K^+^ (KATP) channels, plasma membrane depolarization, and activation of voltage-dependent Ca^2+^ channels. The resulting Ca^2+^ influx into the cytosol serves as the principal trigger for exocytosis of insulin-containing granules [1].

It was shown that glycogen synthase kinase 3 beta (GSK3β) phosphorylates PS1, which, in turn, establishes a constitutive continuous ER Ca^2+^ leak that is transferred to the mitochondria, elevates basal mitochondrial matrix Ca^2+^ levels [2], which stimulates Ca^2+^-sensitive dehydrogenases of the tricarboxylic acid cycle, thereby enhancing oxidative phosphorylation and ATP synthesis and primes the organelle for instant glucose sensing oxidative metabolism, and which is crucial for the initial insulin release [3]. Silencing PS1 or inhibiting GSK3β abolishes this leak, resulting in reduced basal mitochondrial respiration and loss of the first phase of insulin secretion, which contributes to postprandial hyperglycemia and the development of type 2 diabetes mellitus [4].

Although this PS1-mediated ER Ca^2+^ leak is fundamental for β-cell responsiveness and β-cell integrity [5], it raises an important physiological question: how do β-cells maintain ER Ca^2+^ content under continuous ER Ca^2+^ leakage? If such continuous basal ER Ca^2+^ leak is not compensated, the persistent ER Ca^2+^ depletion would impair both mitochondrial metabolism and insulin secretion, indicating that a compensatory Ca^2+^ refilling mechanism must exist. In most cell types, store-operated Ca^2+^ entry (SOCE) through stromal interaction molecule 1 (STIM)—ion channel responsible for store-operated Ca^2+^ entry (ORAI) channels replenishes ER Ca^2+^ stores following agonist/IP3-induced depletion [6,7,8]. However, for activation of SOCE, the ER Ca^2+^ content must be substantially decreased, which would result in a continuous emptying-refilling cycle in β-cells with their established continuous ER Ca^2+^ leak [9,10,11]. As such, the emptying-refilling cycle did not become evident in our previous work, we anticipate a covert mechanism of continuous ER Ca^2+^ refilling as compensation for the continuous ER Ca^2+^ leak in β-cells.

Therefore, this study aimed to identify the plasma membrane transporters and ion channels responsible for covert continuous ER Ca^2+^ refilling in INS-1 β-cells that compensate for PS1-mediated ER Ca^2+^ leak. Hence, we intended to explore the importance of this hidden mechanism for continuous ER Ca^2+^ refilling for mitochondrial bioenergetics and β-cell physiology in pancreatic β-cells. Ultimately, this study should evaluate an unknown Achilles’ heel of β-cell dysfunction in early onset of diabetes when β-cells fail to preserve ER Ca^2+^ balance, cannot compensate for the continuous Ca^2+^ leak established by PS1, and lose glucose sensitivity due to reduced mitochondrial bioenergetics and impaired insulin secretion.

## 2. Materials and Methods

### 2.1. Reagents

KB-R7943 (PubChem ID CID:9823846) and Fluo-4 AM (PubChem ID CID:4060965) were purchased from THP Medical Products (Vienna, Austria). Pyr10 (PubChem ID CID:53475435) and Pyr6 (PubChem ID CID: 10598093) were purchased from Tedubio (Offenbach, Germany). HC-067047 (PubChem ID CID:2742550), oligomycin A (PubChem CID: 5281899), antimycin A (PubChem CID: 16218979), 2,5-di-t-butyl-1, 4-benzohydroquinone (BHQ; PubChem CID: 16043), carbachol (Cch; PubChem CID: 5832), carbonyl cyanide p-trifluoromethoxyphenylhydrazone (FCCP; PubChem CID: 3330), ethylene glycol tetraacetic acid (EGTA; PubChem CID: 6207) were from Sigma Aldrich (Vienna, Austria). Cell culture materials were obtained from Greiner Bio-One (Kremsmünster, Austria). D1ER was from Addgene (Cambridge, MA, USA).

### 2.2. Cell Culture

The immortalized rat insulinoma model cell line (INS-1 832/13) was kindly provided by Prof. Dr. Claes B. Wollheim and Dr. Françoise Assimacopoulos-Jeannet (University Medical Center, Geneva, Switzerland). INS-1 cells were cultured in RPMI 1640 (Thermo Fisher Scientific, Vienna, Austria) containing 11 mM glucose (PubChem CID: 5793) supplemented with 10 mM HEPES (PubChem CID: 23831, Thermo Fisher Scientific, Vienna, Austria), 10% fetal calf serum (FCS, Thermo Fisher Scientific, Vienna, Austria), 1 mM sodium pyruvate (PubChem CID: 23662274, Thermo Fisher Scientific, Vienna, Austria), 50 μM β-mercaptoethanol (PubChem CID: 1567, Thermo Fisher Scientific, Vienna, Austria), 1% (*v*/*v*) Pen Strep^®^ (Thermo Fisher Scientific, Vienna, Austria; 10.000 U/mL). The human beta cell line (EndoC-βH5) was purchased from Human Cell Design, Inc., Toulouse, France, and cultured according to the manufacturer’s guidelines in culture media with 5.5 mM glucose.

### 2.3. Transfection

For Ca^2+^ imaging, cells were seeded on 30 mm glass coverslips placed in 6-well plates and transiently transfected at 60–80% confluency with 0.5 µg of plasmid DNA. Transfection was performed using 3 µL of PolyJet reagent (SignaGen Laboratories, Rockville, MD, USA) in 1 mL of complete culture medium. The cells were then placed in an incubator at 37 °C with 5% CO_2_ and 95% air for 10–12 h, after which the transfection medium was replaced with a full culture medium. All experiments were conducted 40–48 h after transfection or treatment.

For knockdown experiments, cells (60–80% confluence) were transfected either with siRNA or a plasmid combination by using 2.5 µL of ScreenFect A Plus transfection reagent (ScreenFect, Eggenstein-Leopoldshafen, Germany). Afterward, the transfection medium was replaced with 2 mL of full culture medium. Experiments were performed 40–48 h after transfection. Before each experiment, cells were adjusted to room temperature while kept in a storage buffer. Scrambled control siRNA sequence (sense strands, 5′-3′) AGGUAGUGUAAUCGCCUUGTT (siControl), the plasma membrane sodium-calcium exchanger 1 (NCX1) siRNA sequence (sense strands, 5′-3′) CCAUAUAACUGCAGAUACATT (siNCX1), the plasma membrane sodium-calcium exchanger 2 (NCX2) (sense strands, 5′-3′) siRNA sequence AGGCAGCCGAUUAUGATT (siNCX2), the transient receptor potential channel C3 (TRPC3) siRNA sequence (sense strands, 5′-3′) CAUUGGCUAUGUCCUUUAUTT (siTRPC3), the transient receptor potential channel V4 (TRPV4) siRNA sequence (sense strands, 5′-3′) GCUUAACAAUGACGGUCUUTT (siTRPV4), and STIM1 (sense strands, 5′-3′) GGAUCUCAGAGGGAUUUGA (siSTIM1) from Microsynth (Balgach, Switzerland) were used.

### 2.4. mRNA Isolation and PCR Analysis

Total RNA was isolated and processed for reverse transcription, PCR, and real-time PCR according to previously established protocols [12]. The relative mRNA levels of NCX2 and NCX3 were expressed as a fold-difference compared with NCX1 levels. Specific primers for detection and real-time PCR were sourced from Invitrogen (Vienna, Austria), with primer sequences as follows: Rat NCX1 5′-TACGATGACAAGCAGCCACT-3′ and 5′-CTTCCAGCTTGGTGTGTTCG-3′; Rat NCX2 5′-TTCTGCTGTCTGTCATCGAG-3′ and 5′-ACACACACAGCAATGACCAC-3′; Rat NCX3 5′-TTCCTGCATGGTCATCATCC-3′ and 5′-GCCATCCTCAATAGTCTTCC-3′.

### 2.5. Validation of Knock-Down Efficiency

Knock down efficiency was verified by RT-PCR. The RT-PCR primers used are for TRPC3 (forward: TGGGTAACTCAAAGTCCAGGTTAAA; reverse: TGCTGATATCGTGTTGGCTGA), TRPV4 (forward: CCGCGTCCCAAGCAGG; reverse: CCATCACCAGGATCTGCCAT), NCX1 (forward: TACGATGACAAGCAGCCACT; reverse: CTTCCAGCTTGGTGTGTTCG), NCX2 (forward: TTCTGCTGTCTGTCATCGAG; reverse: ACACACACAGCAATGACCAC), and STIM1 (forward: GAAGATGACAGACCGGAGCC; reverse: TTGTGGCGAGTCAAGAGAGG). Individual knockdown efficiency was 42.3 ± 7.5% for TRPC3, 53.6 ± 17.4% for TRPV4, 40.1 ± 0.5% for NCX1, 51.7 ± 1.1% for NCX2, and 52.6 ± 2.1% for STIM1 at an average transfection efficiency between 30–40% (calculated by cells expressing the sensors).

### 2.6. Buffers and Solutions

Before the microscopic measurements, cells were adjusted to room temperature in storage buffer: 2 mM CaCl_2_, 138 mM NaCl, 1 mM MgCl_2_, 5 mM KCl, 10 mM HEPES, 2.6 mM NaHCO_3_, 0.44 mM KH_2_PO_4_, amino acid, and vitamins mix, 10 mM glucose, 2 mM L-glutamine, 1% penicillin/streptomycin, 1.25 μg/mL amphotericin B and pH adjusted to 7.4. Live-cell imaging experiments were performed either in the Hepes-buffered Ca^2+^ containing experimental buffer with normal Na^+^ (2Ca138Na): 2 mM CaCl_2_, 138 mM NaCl, 1 mM MgCl_2_, 5 mM KCl, 10 mM Hepes, and 10 mM D-glucose at pH 7.4 or a nominal Ca^2+^ free solution with normal Na^+^ (0Ca138Na) containing 0.1 mM EGTA or in the Hepes-buffered Ca^2+^ containing experimental buffer with low Na^+^ (2Ca19Na) containing 19 mM NaCl and 119 mM choline chloride.

### 2.7. Live Cell Imaging

Experiments were conducted using an Olympus IX73 inverted microscope equipped with a UApoN340 40× oil immersion objective (Olympus, Tokyo, Japan) and a CCD Retiga R1 camera (Q-imaging, Surrey, BC, Canada). Illumination was provided by LedHUB^®^ (Omicron-Laserage Laserprodukte GmbH, Rodgau-Dudenhofen, Germany) with 340, 385, 455, 470, and 550 nm LEDs combined with a CFP/YFP/RFP (CFP/YFP/mCherry-3X, Semrock, New York, NY, USA) filter set as previously described [13]. Data acquisition was performed using VisiView 4.2.01 (Visitron, Puchheim, Germany). Cells were placed into a flow chamber for the live cell imaging experiments. A gravity-based perfusion system PS-9D (NGFI, Graz, Austria) was used to perfuse cells during measurements. A motorized valve with nine positions was connected to the reservoirs (NGFI, Graz, Austria), and the desired reservoir was automatically activated using perfusion control software (V2, NGFI, Graz, Austria). Cells were randomly selected based on the expression of genetically encoded biosensors.

### 2.8. ER Ca^2+^ Measurement

ER Ca^2+^ levels were measured with a genetically encoded, FRET-based, ER-targeted ratiometric Ca^2+^ sensor D1ER [14] as described previously [15]. This sensor was illuminated with a 455 nm LED every 2 s using a 300-ms exposure time. Emission was collected at 480 nm and 530 nm using a CFP/YFP/mCherry-3X filter set and a 505dcxr beam-splitter. All recordings were background subtracted. Cells were preincubated in the corresponding preincubation buffer for 40 min and then stimulated with 100 µM carbachol (IP_3_-generating agonist) in the presence of 15 µM BHQ (an inhibitor of sarcoplasmic/endoplasmic reticulum calcium ATPase; SERCA) in nominal 0 mM Ca^2+^ 138 mM Na^+^ experimental buffer.

### 2.9. Subplasmalemmal Ca^2+^ Measurements

To measure the subplasmalemmal Ca^2+^ signal, cells were transfected with a genetically encoded Ca^2+^ sensor, GCaMP6s-CAAX [16]. Then, cells were illuminated at 470 nm every 2 s using a 300-ms exposure time, and fluorescence emission was recorded at 510 nm. Cells were perfused with a 2 mM Ca^2+^-containing buffer for 5 min to measure the basal Ca^2+^ oscillations. Subsequently, cells were perfused with the nominal Ca^2+^-free buffer containing 0.1 mM EGTA for 20 min to partially deplete the ER Ca^2+^ content. Then the buffer was replaced with Ca^2+^-containing buffer again to refill the ER Ca^2+^ content and measure the subplasmalemmal Ca^2+^ signal for 5 min. Corresponding inhibitors were used to block the respective channels. All recordings were background subtracted and normalized to the basal level.

### 2.10. Cytosolic Ca^2+^ Measurements

Fluo-4 AM dye was used to measure the cytosolic Ca^2+^ oscillations [17]. Cells were loaded with 2 µM of Fluo-4 AM in a storage buffer with 3 mM glucose instead of 10 mM glucose for 20 min. Then, the cells were washed with the same storage buffer and placed into a glucose-free experimental buffer with DMSO or the corresponding inhibitor for 40–45 min before imaging to minimize the cytosolic Ca^2+^ oscillations. Mannitol was used as a glucose substitute to adjust the osmolarity. On the microscope, cells were perfused with 0 mM glucose (0 G) buffer for 2 min before switching to a 16 mM glucose (16 G) buffer containing the corresponding inhibitors during acquisition. Cells were illuminated at 470 nm every 2 s using a 300-ms exposure time, whereas fluorescence emission was recorded at 510 nm. All recordings were background subtracted. The measurements were normalized to the basal level, and the number of oscillations after glucose stimulation was counted.

Cytosolic Ca^2+^ was also recorded in EndoC-βH5 cells to assess the releasable ER Ca^2+^ pool. Cells were loaded with 2 µM Fluo-4 AM in storage buffer for 20 min at room temperature, washed, and preincubated for 40 min in either the 2 mM Ca^2+^ 138 mM Na^+^ buffer (2Ca138Na) or the nominally Ca^2+^-free 138 mM Na+ buffer containing 0.1 mM EGTA (0Ca138Na). The preincubation buffers contained DMSO as control or one of the following inhibitors: 10 µM KB-R7943, 3 µM Pyr10, 5 µM HC-067047, or 2 µM Pyr6. On the microscope, cells were perfused for 2 min with the corresponding preincubation buffer to record the baseline, after which the ER Ca^2+^ store was depleted with 100 µM carbachol (Cch) and 15 µM BHQ. Cells were illuminated at 470 nm every 2 s using a 300 ms exposure time, and fluorescence emission was recorded at 510 nm. All recordings were background subtracted and normalized to the basal fluorescence (F/F_0_). The peak amplitude of the Cch/BHQ-induced response (Δmax, F/F_0_) was taken as a measure of the releasable ER Ca^2+^ pool.

### 2.11. Mitochondrial Respiration Measurements

INS-1 cells were seeded in XF96 polystyrene cell culture microplates (Seahorse^®^, Agilent, Santa Clara, CA, USA) at a density of 100,000 cells per well. Following overnight incubation, the culture medium was changed to the Seahorse medium with or without inhibitors. The cells were incubated at 37 °C for 1 h and the mitochondrial respiration was analyzed using the XF96 extracellular flux analyzer (Seahorse Bioscience^®^, Agilent, Santa Clara, CA, USA) according to previously described protocols [18].

### 2.12. Insulin Measurements

For the determination of insulin secretion rate INS-1 cells were routinely seeded on 6-well plates. 24 h after seeding, cells were washed twice with HBSS buffer containing 3 mM D-glucose, followed by a 1 h incubation in this buffer with the corresponding inhibitors to record basal insulin secretion. For stimulation of insulin secretion, cells were stimulated with HBSS buffer containing 16 mM D-glucose. Samples were taken at the 10th, 20th, and 45th minutes. Samples were stored on ice and centrifuged at 3000 rpm for 5 min to remove residual cells. Supernatant was transferred to fresh tubes and assayed using the Mercodia Rat Insulin ELISA (Mercodia, Uppsala, Sweden). Insulin secretion rate was calculated as previously described [19] using a standard procedure [20].

### 2.13. Dynamic Measurements of STIM1 Oligomerization

STIM1 oligomerization was analyzed in cells co-expressing CFP-STIM1 and YFP-STIM1 by following FRET dynamics between the two fluorophores as previously described [21].

### 2.14. Statistics

The number of independent experiments and the number of cells, where applicable are indicated in each figure legend along with the statistical test used and significance. Unless otherwise stated, all data are shown as means ± SEM. Microsoft Excel (Microsoft 365, Microsoft, Redmond, WA, USA) was used for the data analysis and calculations. For statistical analysis, such as unpaired Student’s *t*-test or analysis of variance (ANOVA) with Bonferroni post-hoc test where applicable, GraphPad Prism 11 (GraphPad Software, San Diego, CA, USA) was used.

## 3. Results

### 3.1. ER Ca^2+^ Refilling Depends on Plasma Membrane Permeability to Na^+^ and Ca^2+^

To explore the mechanism that compensates for the loss of ER Ca^2+^ due to the PS1-induced ER Ca^2+^ leak, ER Ca^2+^ dynamics were monitored using the ER-targeted Ca^2+^ FRET sensor D1ER [14] in INS-1 cells. During preincubation in standard medium (2 mM Ca^2+^, 138 mM Na^+^), cells maintained stable ER Ca^2+^ content, whereas removing extracellular Ca2^+^ yielded a time-dependent depletion of the ER (Figure 1A, left panel) and decreased the releasable ER Ca^2+^ pool by carbachol (Cch, 100 µM) and BHQ (15 µM) (Figure 1A, right panel).

As these data point to a compensatory mechanism for the PS1-mediated ER Ca^2+^ loss that utilizes extracellular Ca^2+^ for constant ER refilling, we next examined the contribution of TRPC3, TRPV4, or Orai1 in the compensatory mechanisms for ER Ca^2+^ refilling. For this purpose, we developed a simple protocol that allowed us to compare the ER Ca^2+^ content under conditions where compensation of the ER Ca^2+^ leak is in place (in the presence of extracellular Ca^2+^), when no compensation is possible (in the absence of extracellular Ca^2+^), and in the presence of extracellular Ca^2+^ and a respective channel inhibitor for either of these channels.

Inhibition of Orai1 with Pyr6 [22] (2 µM), which was positively tested to prevent store-operated Ca^2+^ entry (Supplementary Figure S1), in the presence of extracellular Ca^2+^ did not affect ER Ca^2+^ dynamics (Figure 1B,E), indicating that refilling occurs independently of classical store-operated Ca^2+^ entry. To further test for the absence of STIM1/Orai1 activation under the experimental conditions used, we have measured STIM1 clustering as previously reported [21]. As shown in Supplementary Figure S2, removal of extracellular Ca^2+^ for 40 min did not trigger STIM1 clustering, while a pronounced ER Ca^2+^ depletion by Cch (100 µM) and BHQ (15 µM) achieved a strong increase in STIM1 clustering. Supportive results were obtained in cells with siRNA-mediated knockdown of STIM1 and STIM2 that did not affect the basal ER Ca^2+^ content (Supplementary Figure S3).

In contrast, inhibition of TRPC3 with Pyr10 [22] (3 µM) and TRPV4 with HC-067047 [23] (5 µM) in the presence of extracellular Ca^2+^ reduced ER Ca^2+^ levels (Figure 1C) and the amount of releasable ER Ca^2+^ pool (Figure 1D). TRPV4 inhibition exerted a stronger effect than TRPC3 (Figure 1E).

Similar findings were found in isolated short-term cultured human pancreatic β-cells (EndoC-βH5), where inhibition of TRPC3 (Pyr10) and TRPV4 (HC-067047), and the reversed mode of NCX (KB-R7943) but not Orai1 (Pyr6) reduced the compensatory ER refilling (Figure 2).

### 3.2. ER Ca^2+^ Refilling Depends on Plasma Membrane Na^+^ Fluxes Fueling NCX Reverse-Mode Activity

Notably, both TRP channels, TRPC3 and TRPV4, are permeable to Na^+^ and Ca^2+^ with a selectivity of PCa:PNa of 1.6 and 6 [24], respectively. Considering the presence of 2 mM Ca^2+^ and 138 mM Na^+^ in our experimental buffer, we speculated that these channels preferentially establish Na^+^ over Ca^2+^ fluxes in beta cells under resting conditions [24].

To further explore the possibility of Na^+^ fluxes as initial compensatory ion movement for the compensation of PS1-mediated ER Ca^2+^ leak, we used the same protocol as in Figure 1, but preincubated the cells in low Na^+^ (19 mM Na^+^) [25] buffer in the presence of extracellular Ca^2+^ for 40 min before ER Ca^2+^ measurement and compared them to the cells preincubated in normal Na^+^ buffer (138 mM Na^+^ 2 mM Ca^2+^). Basal ER Ca^2+^ levels (Figure 3A, left panel) and the amount of releasable ER Ca^2+^ pool (Figure 3A, right panel) were strongly reduced in low extracellular Na^+^ compared with 138 mM controls. These data point to transmembrane Na^+^ fluxes as an important event in the compensation of ER Ca^2+^ loss due to the PS1-established ER Ca^2+^ leak and let us speculate on a joint activity of Na^+^ fluxes established by TRPs and a functionally coupled reverse activity of the plasma membrane Na^+^/Ca^2+^ exchanger (NCX). Therefore, we next tested whether reverse-mode Na^+^/Ca^2+^ exchange [25] contributes to compensating for ER Ca^2+^ loss caused by the PS1-induced ER Ca^2+^ leak. Cells were treated in the presence of extracellular Ca^2+^ with the NCX reverse-mode inhibitor KB-R7943 (10 µM) [26]. KB-R7943 reproduced the effects of low Na^+^ or Ca^2+^ removal and markedly reduced basal ER Ca^2+^ content (Figure 3B) and the amount of releasable ER Ca^2+^ pool (Figure 3B, right panel). Together, these findings suggest that ER Ca^2+^ replenishment for PS1-established ER Ca^2+^ leak in beta cells requires a joint activity of Na^+^ influx via TRPC3 and TRPV4, and the reverse-mode of NCX pumping in Ca^2+^ (Figure 3C).

### 3.3. TRPC3/TRPV4 and NCX Jointly Establish Subplasmalemmal Ca^2+^ Signals for ER Refilling

To further challenge our hypothesis illustrated in Figure 3C, we monitored subplasmalemmal Ca^2+^ signals using the membrane-targeted Ca^2+^ sensor GCaMP6s-CAAX [16]. Cells expressing GCaMP6s-CAAX were perfused with 0 mM Ca^2+^ buffer containing DMSO as a control (Figure 4A), 3 µM Pyr10 (TRPC3 inhibitor) (Figure 4B), or 5 µM HC-067047 (TRPV4 inhibitor) (Figure 4C). After 20 min in a nominal Ca^2+^-free solution, the extracellular buffer was replaced with a 2 mM Ca^2+^ containing buffer without and with the respective inhibitors for TRPC3 and TRPV4, and the subplasmalemmal Ca^2+^ signal was monitored. We expected that in the absence of extracellular Ca^2+^, any Na^+^ currents would be pronounced, yielding subplasmalemmal Na^+^ loading that fuels the NCX upon readdition of extracellular Ca^2+^ to shuttle in Ca^2+^ and increase subplasmalemmal Ca^2+^. Both TRP channel inhibitors strongly reduced the subplasmalemmal Ca^2+^ elevation upon the readdition of extracellular Ca^2+^ (Figure 4), thus supporting the concept that TRPC3 and TRPV4 indeed contribute to subplasmalemmal Na^+^ loading. These data further support the previous findings, indicating that TRPV4 is more involved than TRPC3 in the process of compensating for ER Ca^2+^ loss by PS1-established ER Ca^2+^ leak.

To confirm these findings with pharmacological inhibitors, we employed a genetic approach with siRNA-mediated knockdown of either TRPC3 or TRPV4. An identical experimental protocol to the previous one using cells expressing GCaMP6s-CAAX was performed with cells treated with either an siRNA control or siRNA against TRPC3 or TRPV4. The maximal subplasmalemmal Ca^2+^ elevation upon readdition of extracellular Ca^2+^ was monitored. Knockdown of TRPC3 or TRPV4 (siTRPC3, siTRPV4) recapitulated the effects of the inhibitors, reducing the magnitude of the subplasmalemmal Ca^2+^ elevation upon readdition of extracellular Ca^2+^ (Figure 5A–C,E). Notably, the number of basal subplasmalemmal Ca^2+^ peaks in the presence of extracellular Ca^2+^ (0–5 min) and the subplasmalemmal Ca^2+^ elevation upon readdition of extracellular Ca^2+^ were strongly reduced by the knockdown of TRPC3 and TRPV4 (Figure 5D,E). These results suggest that both TRPC3 and TRPV4 contribute to Na^+^ influx in the presence and absence of extracellular Ca^2+^ conditions and, thus essentially maintain the localized Ca^2+^ microenvironment in the subplasmalemmal area by Na^+^ supply presumably fueling reversed NCX activity [27] for efficient ER Ca^2+^ replenishment.

We next examined the specific transporters responsible for translating the TRP-mediated Na^+^ influx into Ca^2+^ entry. The cells were perfused with 0 mM Ca^2+^ buffer under the microscope, and 2 mM Ca^2+^ containing buffer was introduced with DMSO as a control (Figure 6A), or 10 µM KB-R7943 (Figure 6B), an inhibitor of the reversed mode of NCX, or 2 µM Pyr6, an ORAI1 inhibitor (Figure 6C).

Inhibition of the reversed mode of NCX with KB-R7943 blunted the subplasmalemmal Ca^2+^ refilling signal upon the readdition of extracellular Ca^2+^ after 20 min in Ca^2+^-free solution (Figure 6B,D). These data further support our hypothesis that the reversed mode of NCX translates plasmalemmal Na^+^ fluxes by TRPC3/TRPV4 into Ca^2+^ supply for ER Ca^2+^ refilling to compensate for PS1-induced ER Ca^2+^ leak in pancreatic β-cells (Figure 6E). In contrast, inhibition of Orai1 with Pyr6 had no significant effect on the Ca^2+^ refilling amplitude (Figure 6C,D), reinforcing the model that the classical store-operated Ca^2+^ entry (SOCE) via Orai1 is not the primary driver of basal ER Ca^2+^ refilling in pancreatic β-cells.

Pancreatic β-cells predominantly express the NCX2 isoform over the NCX1 isoform and only very small amounts of NCX3 (Figure 7F). To determine which NCX isoform is involved, we performed isoform-specific knockdown and tested which of the NCX isoforms are involved in the translation of TRPC3/TRPV4-fueled subplasmalemmal Na^+^ to spatial Ca^2+^ elevation. Subplasmalemmal Ca^2+^ was recorded in the presence of extracellular Ca^2+^, followed by 20 min in the nominal absence of extracellular Ca^2+^ (“stimulated Na^+^ loading period”), and the subsequent readdition of 2 mM Ca^2+^ containing buffer. The number of subplasmalemmal Ca^2+^ oscillations during the basal conditions in the presence of extracellular Ca^2+^ (0–5 min), and the maximal subplasmalemmal Ca^2+^ elevation upon the Ca^2+^ readdition were measured. Knockdown of NCX1 did not affect basal Ca^2+^ oscillations or the Ca^2+^ peak upon Ca^2+^ readdition (Figure 7). In contrast, knockdown of NCX2 strongly reduced the number of basal Ca^2+^ peaks and the subplasmalemmal Ca^2+^ amplitude (Figure 7).

### 3.4. TRPC3, TRPV4, and the Reversed Mode of NCX Jointly Establish Glucose-Induced Cytosolic Ca^2+^ Oscillations in Pancreatic β-Cells

To assess the functional significance of this complex Na^+^/Ca^2+^ signaling in β-cells, glucose-triggered cytosolic Ca^2+^ oscillations were recorded using Fluo-4 AM [17]. β-cells were incubated for 40 min with the various inhibitors or DMSO as a control, and subsequently stimulated with 16 mM glucose. Control cells displayed a regular number of oscillations, while inhibition of NCX reverse mode (KB-R7943), TRPC3 (Pyr10), or TRPV4 (HC-067047), markedly reduced the number of oscillations (Figure 8A–E). Quantification revealed the strongest reduction upon NCX inhibition > TRPV4 > TRPC3, indicating that the TRPV4/TRPC3–NCX contributes critically to maintaining glucose-induced Ca^2+^ oscillations in β-cells.

### 3.5. NCX Reverse Mode, TRPC3, and TRPV4 Jointly Contribute to Mitochondrial Oxygen Consumption

Because ER–mitochondria Ca^2+^ transfer is a key driver of basal β-cell oxidative metabolism [28,29,30], we investigated the impact of NCX reverse mode, TRPC3, and TRPV4 on basal mitochondrial respiration. β-cells were preincubated with or without the respective inhibitors for 40 min before the experiment. Seahorse^®^ analysis revealed that inhibition of NCX reverse mode (KB-R7943), TRPC3 (Pyr10), or TRPV4 (HC-067047) significantly decreased both basal and maximal oxygen consumption rates (OCR) compared with control cells (Figure 9). These results indicate that TRPC3/TRPV4–NCX-mediated ER Ca^2+^ refilling supports basal mitochondrial Ca^2+^ uptake and respiration, linking ER Ca^2+^ homeostasis directly to the ER Ca^2+^ leak and β-cell basal metabolic activity.

### 3.6. TRPC3/TRPV4, Jointly with NCX, Ensure First-Phase Insulin Secretion upon Elevated Glucose

Finally, we examined whether interference with the compensation for the PS1-mediated ER Ca^2+^ leak affects glucose-stimulated insulin secretion (GSIS). Therefore, β-cells were incubated in 3 mM glucose-containing medium with or without the corresponding inhibitors for 1 h, followed by a shift to 16 mM glucose with or without the corresponding inhibitors. The secreted insulin was measured at 0, 10, 20, and 45 min after the glucose elevation. In control β-cells, glucose stimulation (16 mM) evoked the already described biphasic insulin release (Figure 10).

Inhibition of NCX, TRPC3, or TRPV4 strongly reduced first-phase insulin secretion (Figure 10A,C), whereas effects on the second phase were only moderate (Figure 10B,D). As in most experiments illustrated above, TRPV4 inhibition produced the strongest suppression of insulin release. These results support our hypothesis of a joint action of TRPV4 > TRPC3 for subplasmalemmal Na+ that is translated to Ca^2+^ uptake via a reversed mode of NCX2 to compensate for PS1-established ER Ca^2+^ leak, thus maintaining ER Ca^2+^ content, and β-cell sensitivity to elevated glucose to ensure adequate GSIS.

## 4. Discussion

Pancreatic β-cells rely on tightly regulated Ca^2+^ fluxes to couple glucose metabolism to insulin secretion [31,32]. A continuous ER Ca^2+^ leak, established by presenilin-1 (PS1) [2,3], requires an equally continuous Ca^2+^ refilling mechanism to preserve ER Ca^2+^ content that essentially maintains mitochondrial metabolism, cytosolic Ca^2+^ oscillations, and glucose-stimulated insulin secretion. Here, we identify a subplasmalemmal Na^+^-dependent TRPC3/TRPV4—NCX2 Ca^2+^ entry pathway that constitutively replenishes ER Ca^2+^ in INS-1 β-cells. We show that at least two plasma membrane TRP channels participate in this process. Our data that pharmacological inhibition of TRPC3 with Pyr10 [22] and TRPV4 with HC-067047 [23], as well as protein-specific siRNA-mediated knockdown, significantly reduced the Ca^2+^ signal in the subplasmalemmal region during ER refilling and decreased ER Ca^2+^ refilling, point to a contribution of these TRP channels in the compensatory mechanism for the PS1-established ER Ca^2+^ leak in resting β-cells. Such basal activity in resting cells is consistent with the reported biophysical properties of these very TRP channels. In this respect, electrophysiological studies have described native TRPC3 activity, thereby establishing a constitutively active channel that maintains spontaneous activity at rest in rabbit ear artery myocytes [33]. Similarly, TRPV4 functions as a metabolic sensor in enteroendocrine cells, where it is activated by glucose to regulate GLP-1 release [34]. While our data focus on their constitutive role in the compensation of PS1-established ER Ca^2+^ leak for maintaining ER Ca^2+^, the established sensitivity of these channels to nutrient cues—such as TRPC3 activation by lipid-derived diacylglycerol (DAG) [35] and TRPV4 modulation by glucose-induced metabolic shifts [34]—provides a compelling framework for their function in pancreatic β-cells. These characteristics suggest a model in which TRPC3 and TRPV4 serve as dynamic metabolic integrators, potentially linking fluctuations in the local nutrient environment to the maintenance of β-cell basal Ca^2+^ homeostasis that is vital for the cell’s responsiveness to elevated glucose and associated insulin secretion [36]. This proposed correlation suggests that the ER refilling rate could scale with the β-cell’s metabolic load, ensuring that Ca^2+^ stores are replenished more efficiently during periods of high demand.

Our results showing that extracellular Na^+^ is fundamental for maintaining ER Ca^2+^ under conditions of PS1-mediated ER Ca^2+^ leak suggest that TRPC3 and TRPV4 preferentially establish Na^+^ conductance under resting conditions. This assumption is in line with the reported selectivity of these channels for Na^+^ and Ca^2+^ (PCa:PNa of 1.6 and 6, for TRPC3 and TRPV4, respectively) [24] at the given intra- and extracellular ion ratios and resting membrane potential. In line with these findings, inhibition of the NCX reverse mode with KB-R7943 strongly reduced ER Ca^2+^ levels under basal conditions of PS1-evoked ER Ca^2+^ leak. In contrast, inhibition of ORAI1 with Pyr6 had no significant effect on basal ER Ca^2+^ refilling. These findings support a model for the basal compensatory mechanism of the PS1-induced ER Ca^2+^ leak in resting pancreatic β-cells (Figure 6E) to rely on TRPC3 and TRPV4 basal Na^+^ conductances, which, in turn, fuels the reversed mode of NCX, ultimately achieving continuous ER Ca^2+^ refilling under these conditions, while no activation of STIM/ORAI1 occurs [27]. This assumption was further supported by our findings that, under our experimental conditions of an impossible overt ER Ca^2+^ depletion, no STIM1 clustering was found within 40 min in the absence of extracellular Ca^2+^. In contrast, Cch/BHQ strongly triggered STIM1 clustering in the same cell. These findings may indicate that the depletion of the ER due to the PS1-established Ca^2+^ leak is too small to activate STIM1 clustering. Alternatively, β cells may contain diverse configurations of ER compartments in which the TRPC3/TRPV4-NCX system may function in discrete ER segments (e.g., closely opposed to the plasma membrane), while not predominating in other ER spatial segments.

Such physical and functional assembly of a TRP-NCX signaling complex is well-documented in other tissues. In cardiac myocytes, TRPC3 is physically associated with NCX1, where PLC-mediated Na^+^ loading through TRPC3 drives NCX into reverse mode to control Ca^2+^ homeostasis [27]. A functional interaction with NCX is not limited to TRPC3; in airway smooth muscle, TRPV4 has been shown to interact directly with NCX and IP3R1 to modulate Ca^2+^ signaling and muscle tension [37].

The use of a Na^+^-dependent “double relay” from TRP-mediated Na^+^ influx to NCX-mediated Ca^2+^ entry might represent a strategic evolutionary adaptation for spatial ion compartmentalization in specialized cells. According to this model, by using Na^+^ as a signaling intermediary, the pancreatic β-cell may control selectively isolated Ca^2+^ movements within the subplasmalemmal-ER junction. This would allow the entering Ca^2+^ to be immediately sequestered by SERCA to refill the ER, effectively “buffering” the signal and preventing it from reaching the global cytosol where it might otherwise trigger unscheduled insulin vesicle exocytosis. While the aforementioned studies often implicate NCX1, we identified the specific involvement of the NCX2 isoform in this β-cell model. This finding suggests a specialized role for NCX2 in maintaining beta-cell Ca^2+^ levels. Accordingly, our proposed mechanism posits that Na^+^ influx through TRPC3/TRPV4 creates the microdomain necessary to flip NCX2 into reverse mode, allowing Ca^2+^ entry that is subsequently sequestered by SERCA to refill the ER (Figure 6E).

Functionally, inhibiting the joined TRPC3/TRPV4 and NCX2 compensated for PS1-established ER Ca^2+^ leak, mimicked the PS1 knockdown phenotype in this cell type, and impaired glucose-induced cytosolic Ca^2+^ oscillations, mitochondrial oxygen consumption, and first-phase insulin secretion at elevated glucose.

Interestingly, inhibition of TRPC3 by Pyr10 leads to increased insulin secretion in the second phase. We cannot rule out that Pyr10 by itself and independently of the inhibition of TRPC3 affects the second phase of insulin secretion. Considering that TRPC3 knockout has been reported to decrease glucose-induced insulin secretion [38], this possibility appears reasonable. Alternatively, the inhibition of TRPC3 might favor a considerably more direct Ca^2+^ influx via TRPV4, which has a superior Ca^2+^ to Na^+^ permeability compared with TRPC3 (6 vs. 1.6) [24]. Consequently, this Ca^2+^ influx might exceed the overt subplasmalemmal Ca^2+^ increase and support the L-type channel-triggered Ca^2+^-mediated fusion of insulin-carrying vesicles with the plasma membrane in the second phase of the response. Alternatively, the enhanced subplasmalemmal Ca^2+^ accumulation by TRPV4 might be exchanged for Na^+^ by the forward mode of NCX, while the increased Na^+^ promotes insulin release [39].

These findings are confirmed by reports that TRPC3 is functionally expressed in islet β-cells, where its activation enhances insulin secretion and its deletion leads to glucose intolerance [37]. Moreover, pharmacological activation of TRPV4 has been shown to modulate Ca^2+^ levels in INS-1 cells, reinforcing its role in β-cell Ca^2+^ homeostasis [38]. Notably, TRPV4 triggers insulin secretion [40], while TRPV4 knockout is protective for pancreatic beta-cells [41]. The underlying mechanisms are not completely understood and may be due to metabolic consequences of TRPV4 knockout. Alternatively, continuous activation of TRPV4 under certain conditions may lead to mitochondrial Ca^2+^ overload leading to ER and cell dysfunction. Altogether, these data indicate an involvement of TRPC3 and TRPV4 in beta cell ER Ca^2+^ homeostasis in our model systems (INS1 and isolated short-term cultured human pancreatic beta cells), their individual role might differ depending on the species and/or the individual metabolic situation.

## 5. Conclusions

Collectively, our present data support the hypothesis that the TRPC3/TRPV4–NCX2 module is required to sustain the ER-mitochondria Ca^2+^ flux content compensating for PS1-established ER Ca^2+^ leak in pancreatic β-cells. This “covert” refilling mechanism ensures that mitochondria are bioenergetically primed to maintain the proper reactivity of the Ca^2+^-sensitive dehydrogenases of the TCA cycle [42,43] and the Ca^2+^-dependent malate-aspartate shuttle [44] in the first phase of a glucose elevation. The present findings further provide information on novel potential targets contributing to β-cell dysfunction, as an impairment of the compensation mechanism of the ER Ca^2+^ leak, leading to reduced metabolic activation and defective GSIS (Figure 10E).

## Figures and Tables

**Figure 1:**
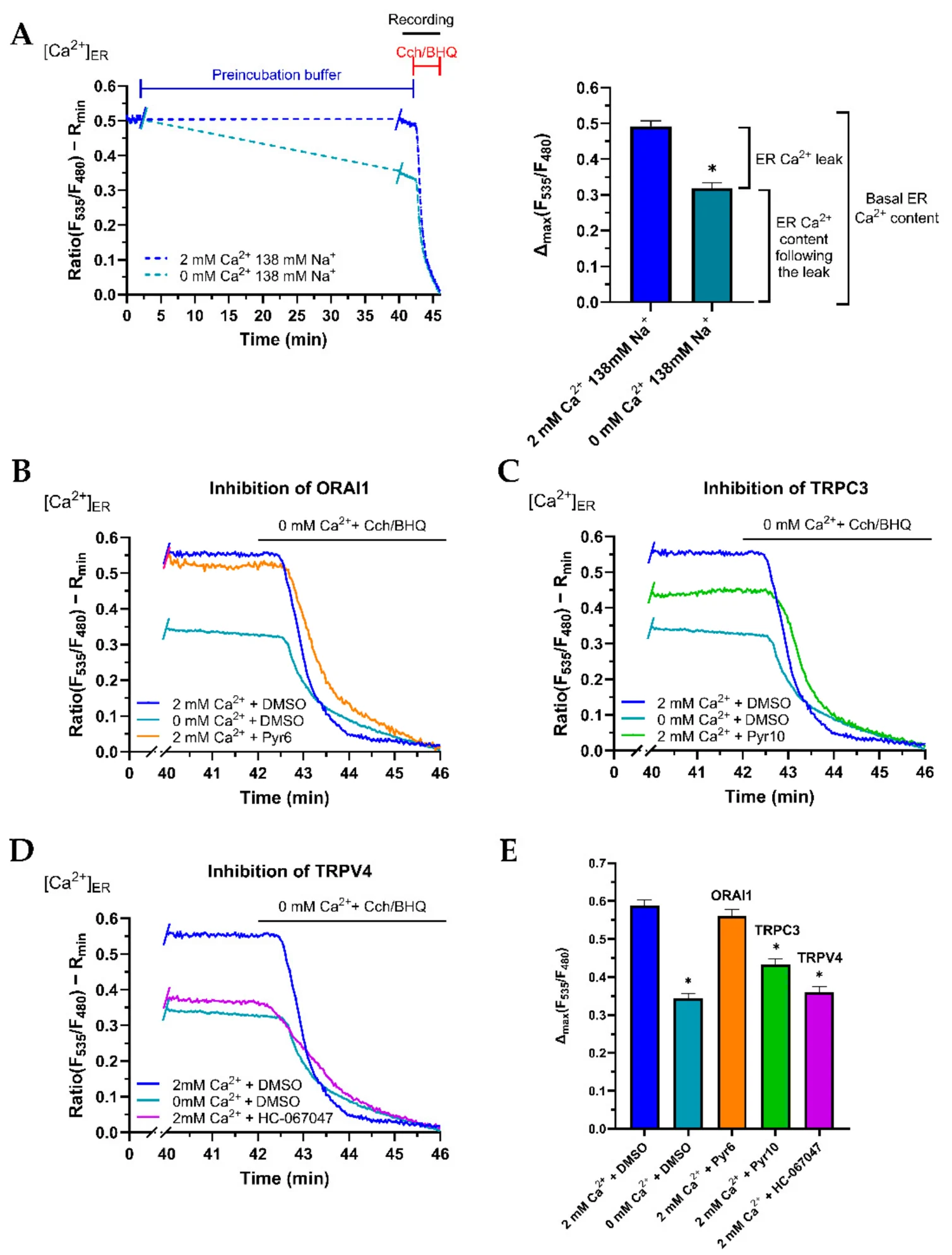
ER Ca^2+^ refilling depends on extracellular Ca^2+^ and the inhibition of TRPC3, TRPV4, but not ORAI1, reduces ER Ca^2+^ refilling. (**A**) Left panel: Representative D1ER FRET traces showing endoplasmic reticulum (ER) Ca^2+^ dynamics during preincubation in 2 mM Ca^2+^ 138 mM Na^+^ (dark blue dashed line) or 0 mM Ca^2+^ 138 mM Na^+^ (cyan dashed line). Right panel: Bar graphs show the ER Ca^2+^ content after 40 min of preincubation in 2 mM Ca^2+^ (blue) and 0 mM Ca^2+^ (cyan). Data points represent the mean ± SEM (n for 2 mM = 53/6; n for 0 mM = 50/6). Significant differences were assessed with an unpaired Student’s *t*-test (* *p* < 0.05). (**B**) Average D1ER FRET traces showing ER Ca^2+^ dynamics in 2 mM Ca^2+^ + DMSO (blue), 0 mM Ca^2+^ + DMSO (cyan), and 2 mM Ca^2+^ + Pyr6 (orange). (**C**) Average D1ER FRET traces showing ER Ca^2+^ dynamics in 2 mM Ca^2+^ + DMSO (blue), 0 mM Ca^2+^ + DMSO (cyan), and 2 mM Ca^2+^ + Pyr10 (green). (**D**) Average D1ER FRET traces showing ER Ca^2+^ dynamics in 2 mM Ca^2+^ + DMSO (blue), 0 mM Ca^2+^ + DMSO (cyan), and 2 mM Ca^2+^ + HC-067047 (purple). (**E**) Bar graphs show the summary quantification of ER Ca^2+^ content after 40 min of preincubation with indicated inhibitors. Data points represent mean ± SEM (n for 2 mM Ca^2+^ + DMSO = 42/6; n for 0 mM Ca^2+^ + DMSO = 67/6; n for 2 mM Ca^2+^ + Pyr10 = 86/6; n for 2 mM Ca^2+^ + HC-067047 = 83/6; n for 2 mM Ca^2+^ + Pyr6 = 59/6). Significant differences were assessed using one-way ANOVA with Bonferroni post hoc test (* *p* < 0.05 versus control).

**Figure 2:**
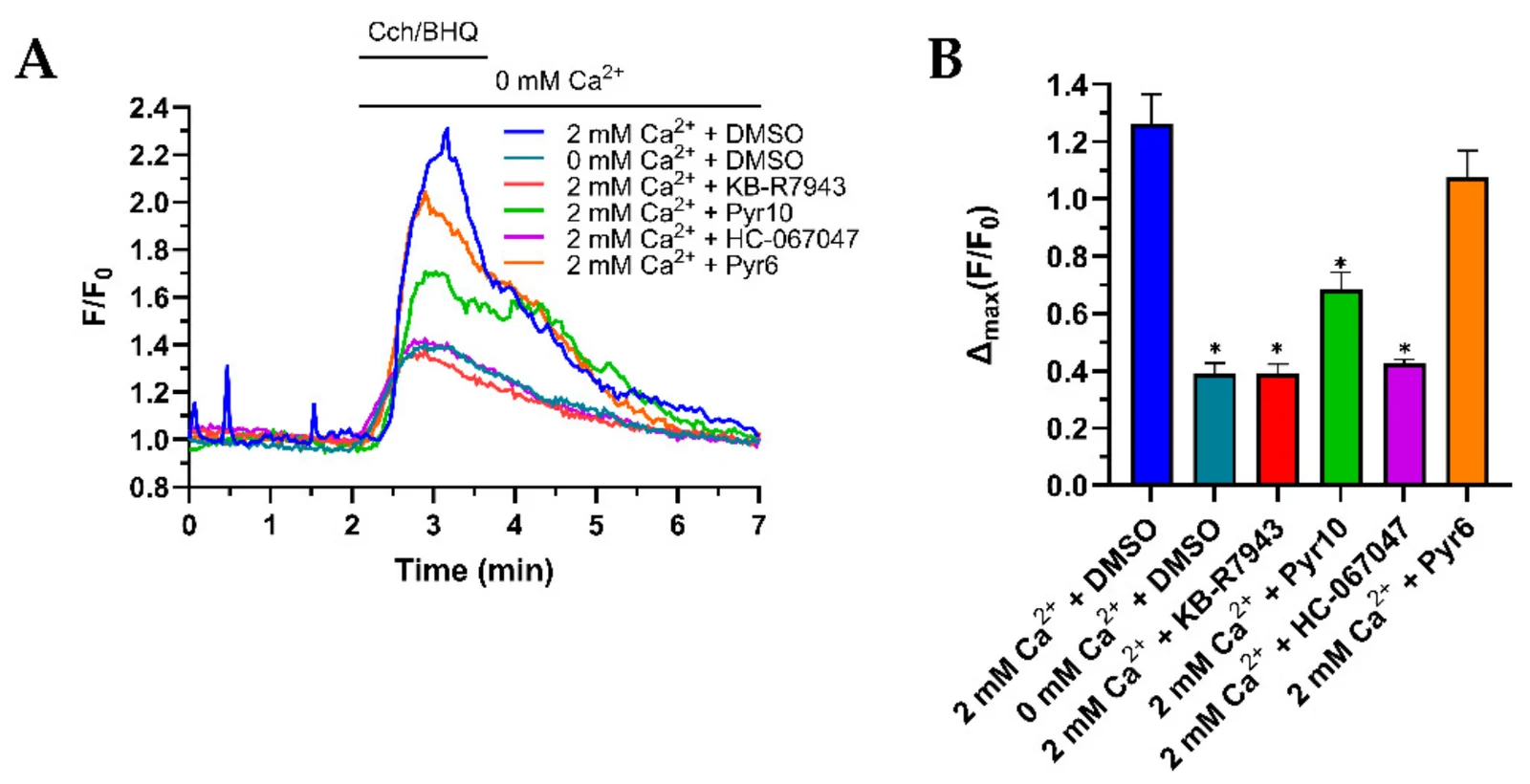
Inhibition of TRPC3, TRPV4, and NCX reverse mode, but not ORAI1, reduces cytosolic Ca^2+^ entry after store depletion in EndoC-βH5 cells. (**A**) Representative Fluo-4 AM traces showing cytosolic Ca^2+^ ([Ca^2+^]_cyto_, F/F_0_) over time in EndoC-βH5 cells preincubated for 40 min in the respective buffer (2 mM Ca^2+^ + DMSO, dark blue; 0 mM Ca^2+^ + DMSO, cyan; 2 mM Ca^2+^ + KB-R7943, red; 2 mM Ca^2+^ + Pyr10, green; 2 mM Ca^2+^ + HC-067047, purple; 2 mM Ca^2+^ + Pyr6, orange). Cells were stimulated with 100 µM carbachol (Cch) and 15 µM BHQ to deplete ER Ca^2+^ stores. The 0 mM Ca^2+^ + DMSO condition was kept in Ca^2+^ free solution throughout, isolating the Ca^2+^ release component, whereas all other conditions retained 2 mM extracellular Ca^2+^ throughout, allowing concurrent Ca^2+^ entry. (**B**) Bar graph shows the peak amplitude of the cytosolic Ca^2+^ rise (Δmax, F/F0) for each condition. Data represent mean ± SEM (n for 2 mM Ca^2+^ + DMSO = 35/3; 0 mM Ca^2+^ + DMSO = 48/3; 2 mM Ca^2+^ + KB-R7943 = 43/3; 2 mM Ca^2+^ + Pyr10 = 32/3; 2 mM Ca^2+^ + HC-067047 = 43/3; 2 mM Ca^2+^ + Pyr6 = 42/3). Significant differences were assessed using one-way ANOVA with Bonferroni post hoc test (* *p* < 0.05 versus 2 mM Ca^2+^ + DMSO control).

**Figure 3:**
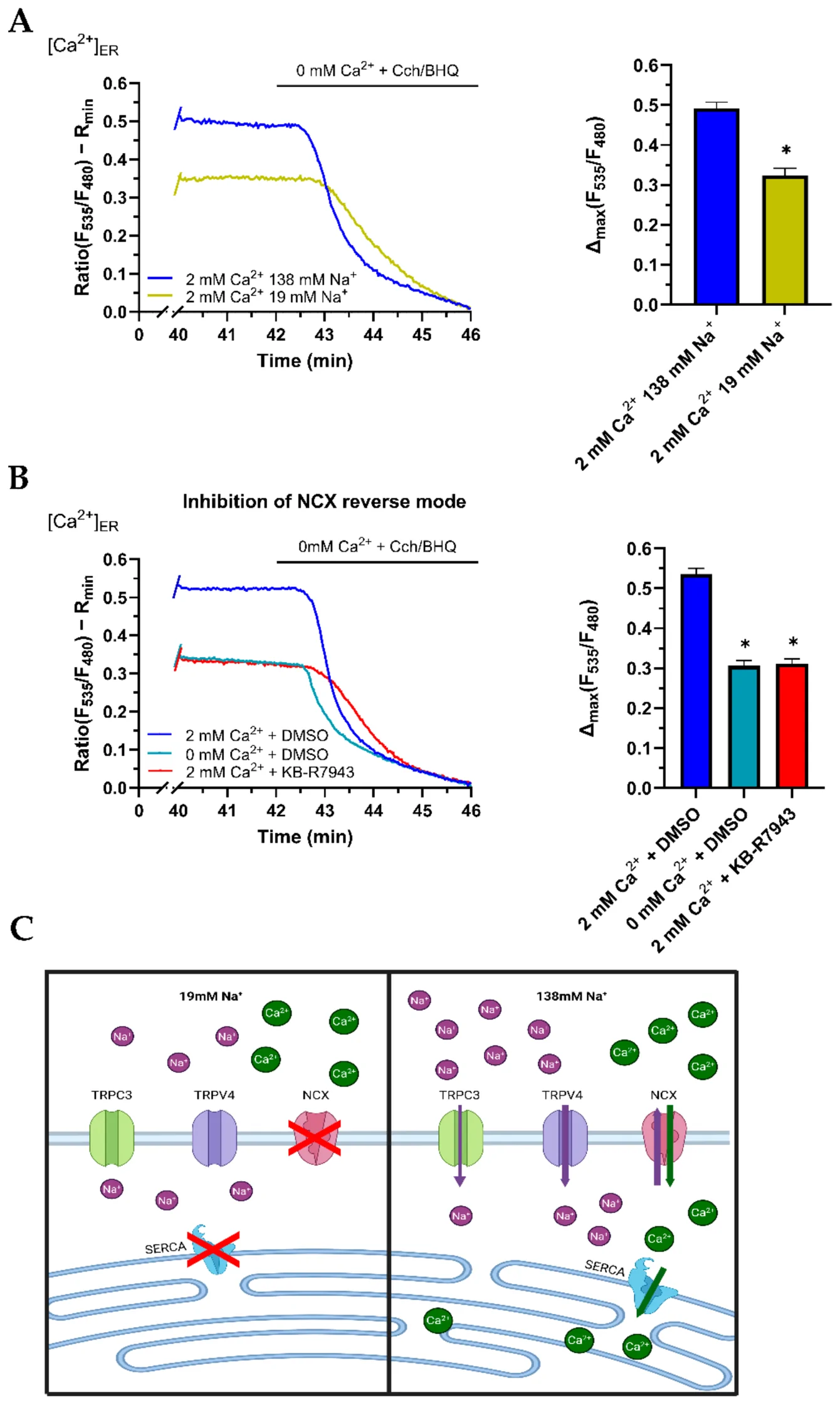
ER Ca^2+^ refilling depends on extracellular Na^+^ and NCX reverse mode activity. (**A**) Left panel: Average D1ER FRET traces showing ER Ca^2+^ dynamics in 138 mM Na^+^ (blue) or 19 mM Na^+^ (yellow). Right panel: Bar graphs show the ER Ca^2+^ content after 40 min of preincubation. Data points represent mean ± SEM (n for 138 Na = 53/6; n for 19 Na = 42/6). (**B**) Left panel: Average D1ER FRET traces showing ER Ca^2+^ dynamics in 2 mM Ca^2+^ + DMSO (blue), 0 mM Ca^2+^ + DMSO (cyan), and 2 mM Ca^2+^ + KB-R7943 (red). Right panel: Bar graphs show the ER Ca^2+^ content after preincubation. Data points represent mean ± SEM (n for 2 mM Ca^2+^ + DMSO = 53/6; n for 0 mM Ca^2+^ + DMSO = 50/6; n for 2 mM Ca^2+^ + KB-R7943 = 61/6). Significant differences were assessed with unpaired Student’s *t*-test for (**A**) and one-way ANOVA for (**B**) (* *p* < 0.05). (**C**) Schematic representation of the proposed mechanism. Left: at low extracellular Na^+^ (19 mM), NCX reverse mode and SERCA-mediated ER refilling are prevented (red crosses). Right: at 138 mM Na^+^, Na^+^ influx through TRPC3 and TRPV4 (purple arrows) drives Ca^2+^ entry via reverse-mode NCX (green arrows), and SERCA uses this Ca^2+^ to refill the ER.

**Figure 4:**
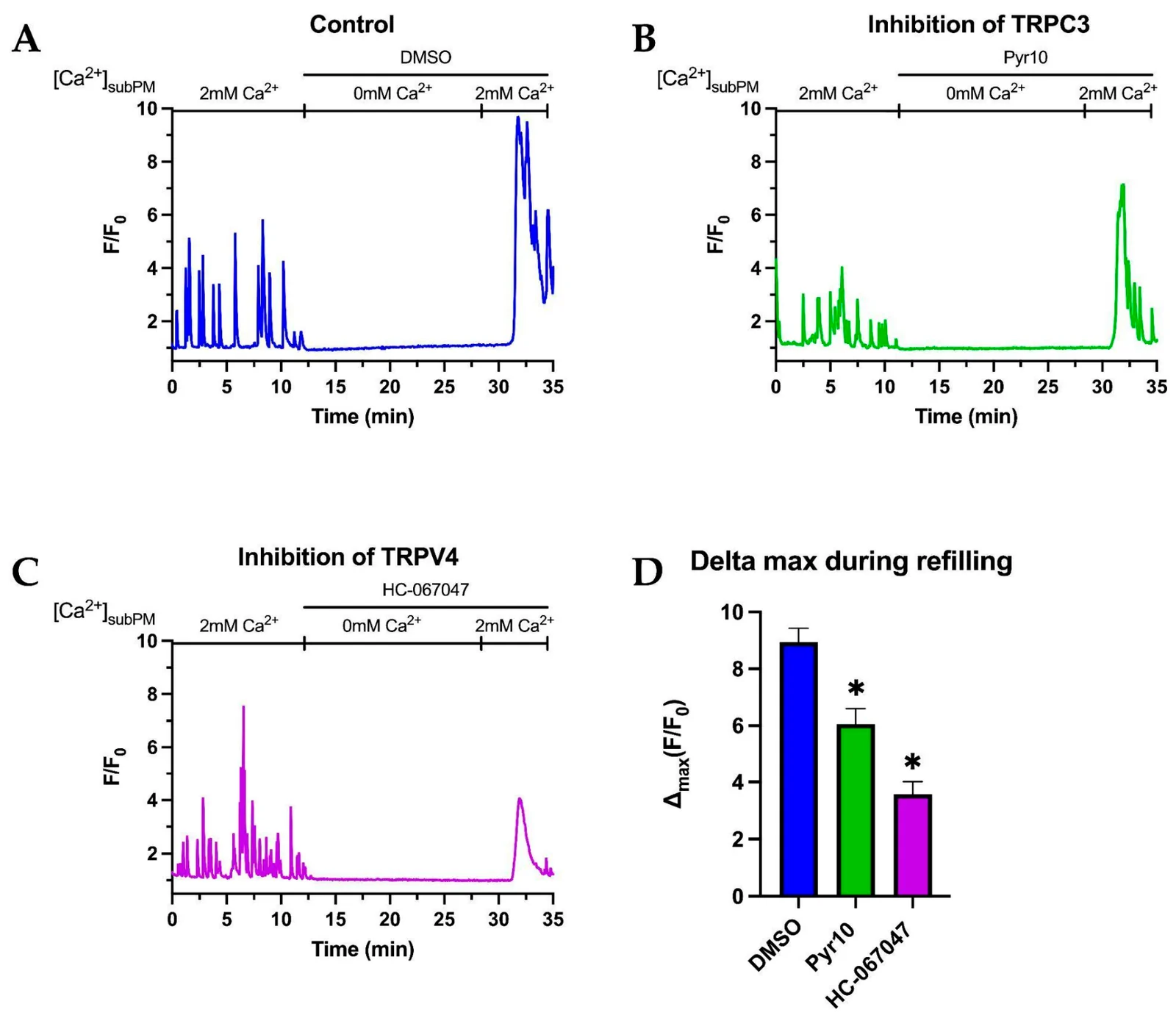
Inhibition of TRPC3 and TRPV4 reduces Ca^2+^ signal in subplasmalemmal region after partial ER depletion in 0 mM Ca^2+^. Representative temporal traces of subplasmalemmal Ca^2+^ concentration expressed as fluorescence intensity normalized to the basal (F/F0) in (**A**) DMSO, (**B**) Pyr10, and (**C**) HC-067047. Protocols indicate transitions from 2 mM to 0 mM Ca^2+^ followed by Ca^2+^ restoration. (**D**) Bar graphs show the maximum Ca^2+^ amplitude during the refilling phase. Data points represent mean ± SEM (n for DMSO = 68/6; n for Pyr10 = 92/6; n for HC-067047 = 121/6). Significant differences were assessed using one-way ANOVA with Bonferroni post hoc test (* *p* < 0.05 versus control).

**Figure 5:**
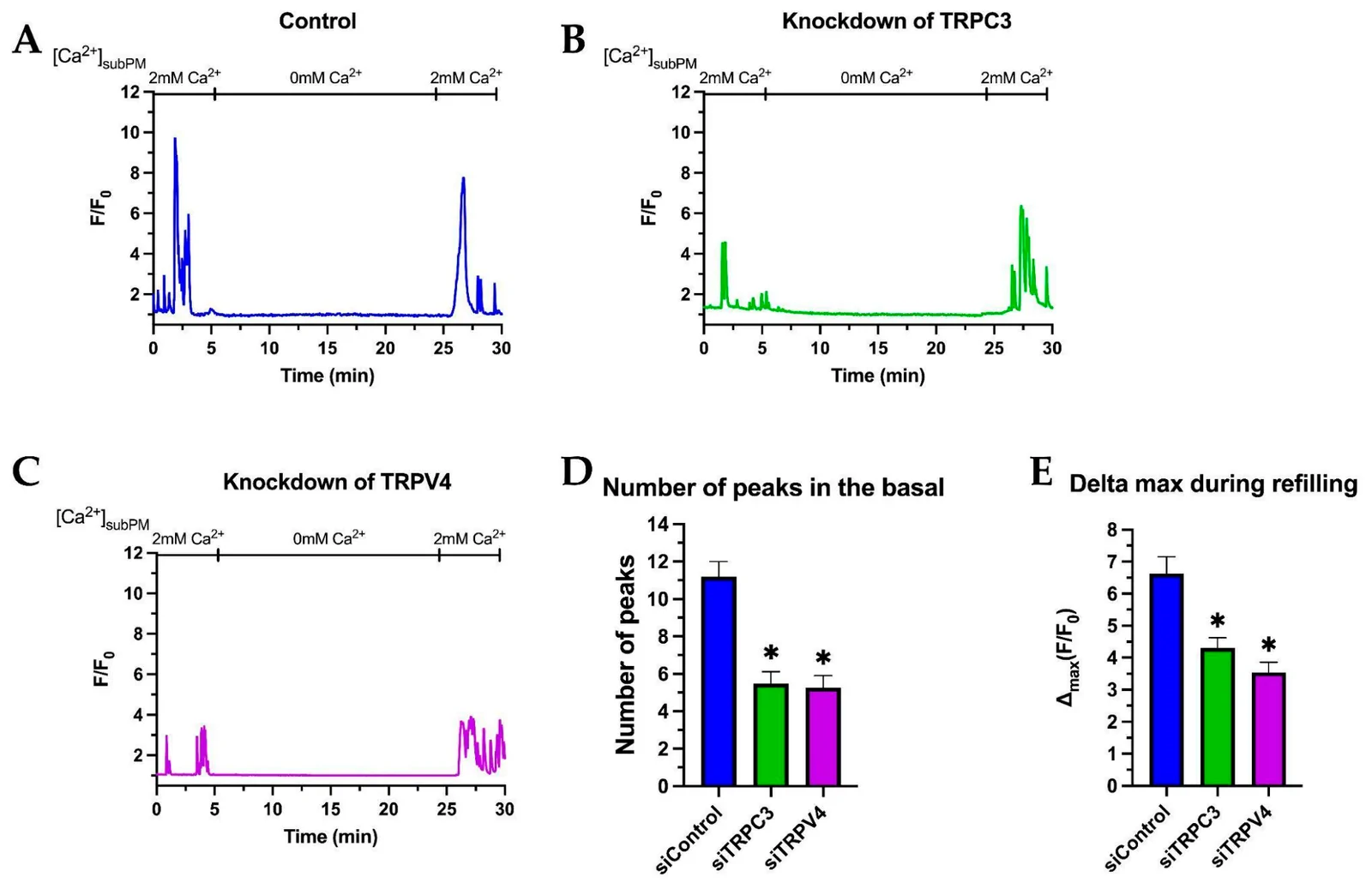
Knockdown of TRPC3 and TRPV4 reduces Ca^2+^ signal in subplasmalemmal region after partial ER depletion in 0 mM Ca^2+^. Representative temporal traces of subplasmalemmal Ca^2+^ concentration expressed as fluorescence intensity normalized to the basal (F/F0) in cells transfected with (**A**) siControl, (**B**) siTRPC3, or (**C**) siTRPV4. (**D**) Bar graphs show the number of Ca^2+^ oscillations in the basal state and (**E**) the maximum Ca^2+^ amplitude during the refilling phase. Data points represent mean ± SEM (n for siControl = 109/6; n for siTRPC3 = 90/6; n for siTRPV4 = 98/6). Significant differences were assessed using one-way ANOVA with Bonferroni post hoc test (* *p* < 0.05 versus control).

**Figure 6:**
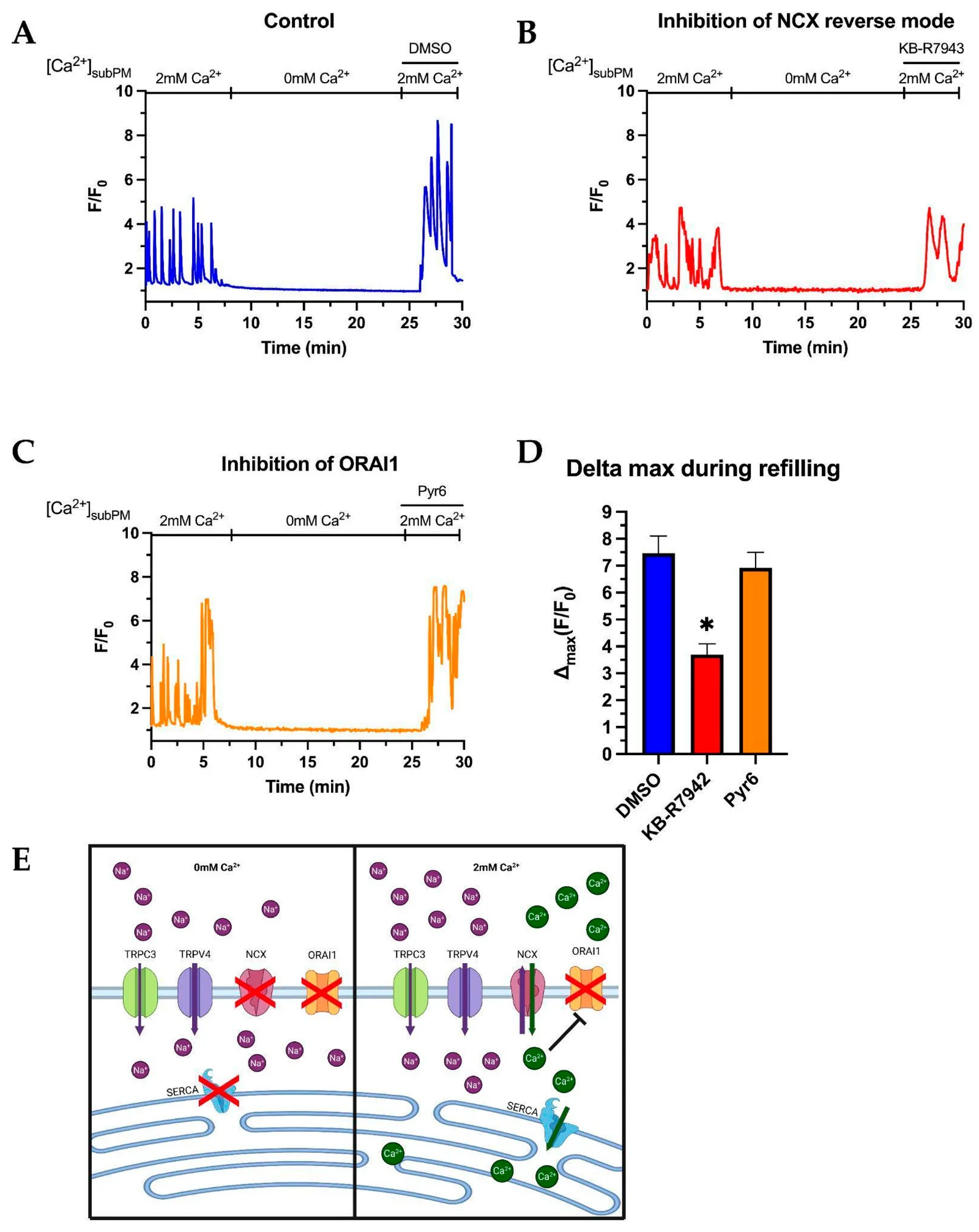
Inhibition of NCX reverse mode, but not ORAI1, reduces Ca^2+^ signal in subplasmalemmal region after partial ER depletion in 0 mM Ca^2+^. Representative temporal traces of subplasmalemmal Ca^2+^ concentration expressed as fluorescence intensity normalized to the basal (F/F0) in (**A**) Control (DMSO), (**B**) KB-R7943, and (**C**) Pyr6-treated cells. (**D**) Bar graphs show the maximum Ca^2+^ amplitude during the refilling phase. Data points represent mean ± SEM (n for DMSO = 115/6; n for KB-R7943 = 91/6; n for Pyr6 = 83/6). Significant differences were assessed using one-way ANOVA with Bonferroni post hoc test (* *p* < 0.05). (**E**) Schematic illustration of the TRPC3/TRPV4 and NCX reverse mode pathway compared with ORAI1 inhibition.

**Figure 7:**
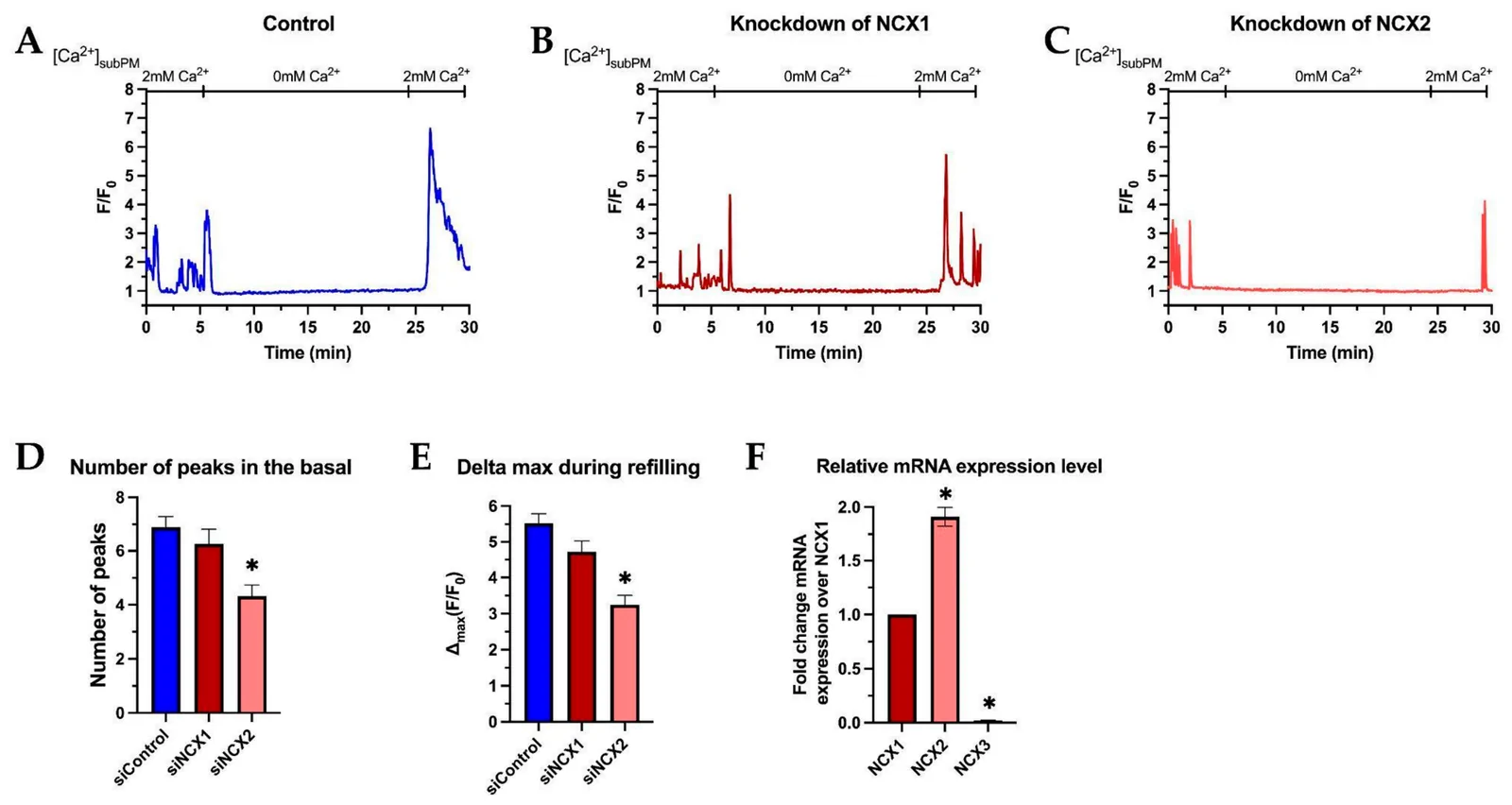
Knockdown of NCX2, but not NCX1, reduces Ca^2+^ signal in subplasmalemmal region after partial ER depletion in 0 mM Ca^2+^. Representative temporal traces of subplasmalemmal Ca^2+^ concentration expressed as fluorescence intensity normalized to the basal (F/F0) in cells transfected with (**A**) siControl, (**B**) siNCX1, or (**C**) siNCX2. (**D**) Bar graphs show the number of Ca^2+^ oscillations in the basal state and (**E**) the maximum Ca^2+^ amplitude during the refilling phase. Data points represent mean ± SEM (n for siControl = 177/6; n for siNCX1 = 121/6; n for siNCX2 = 144/6). (**F**) Relative expression level of NCX1, NCX2 and NCX3 in INS1 cells (*n* = 9/3). Significant differences were assessed using one-way ANOVA with Bonferroni post hoc test (* *p* < 0.05 versus control).

**Figure 8:**
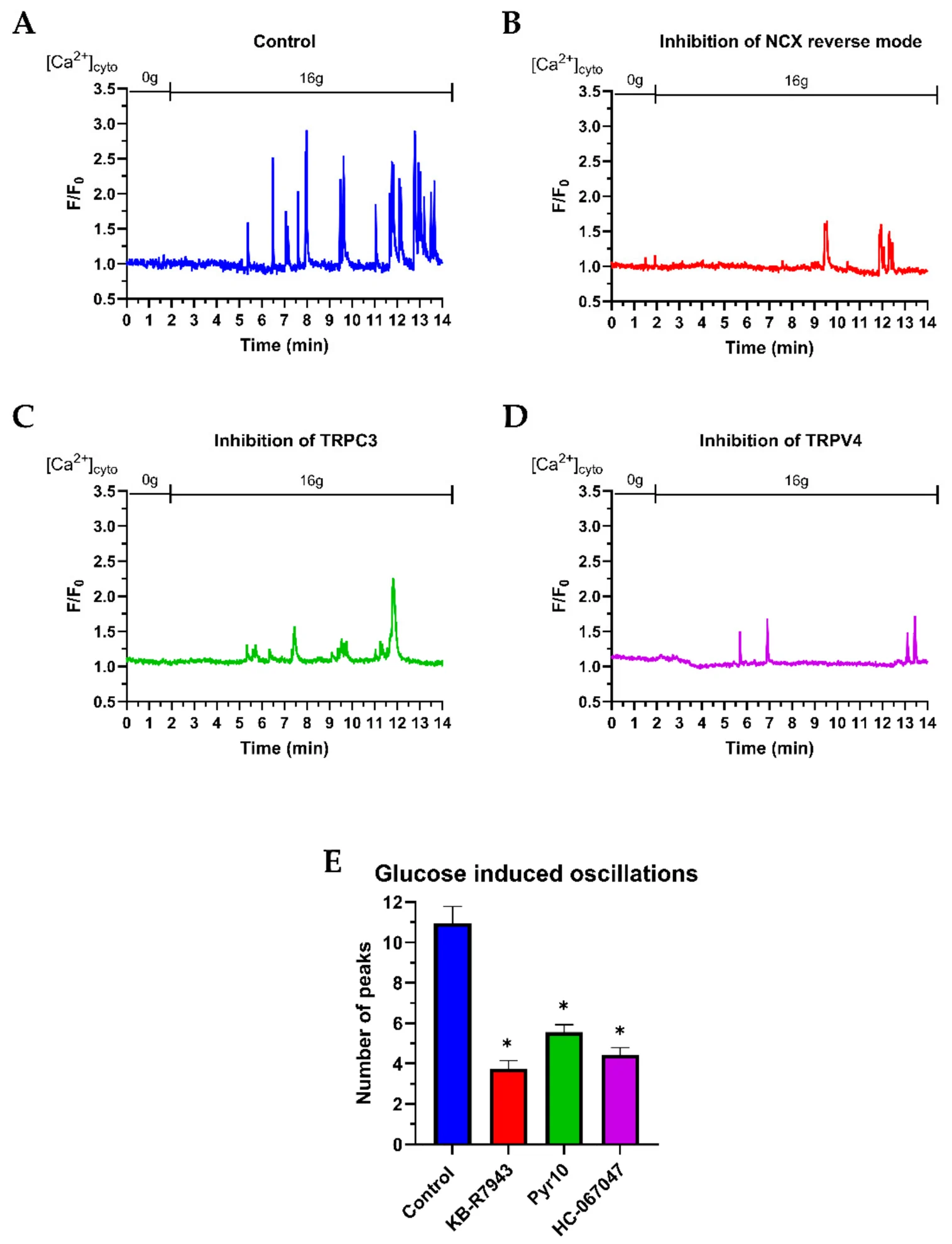
NCX and TRP channels are required to maintain glucose-induced cytosolic Ca^2+^ oscillations. Representative cytosolic Ca^2+^ traces measured with Fluo-4 AM in INS-1 cells stimulated with 16 mM glucose under (**A**) control (blue), (**B**) KB-R7943 (red), (**C**) Pyr10 (green), and (**D**) HC-067047 (purple) conditions. (**E**) Bar graphs show the number of Ca^2+^ oscillations number during glucose stimulation. Data points represent mean ± SEM (n for Control = 164/6; n for KB-R7943 = 261/6; n for Pyr10 = 279/6; n for HC-067047 = 271/6). Significant differences were assessed using one-way ANOVA with Bonferroni post hoc test (* *p* < 0.05 versus control).

**Figure 9:**
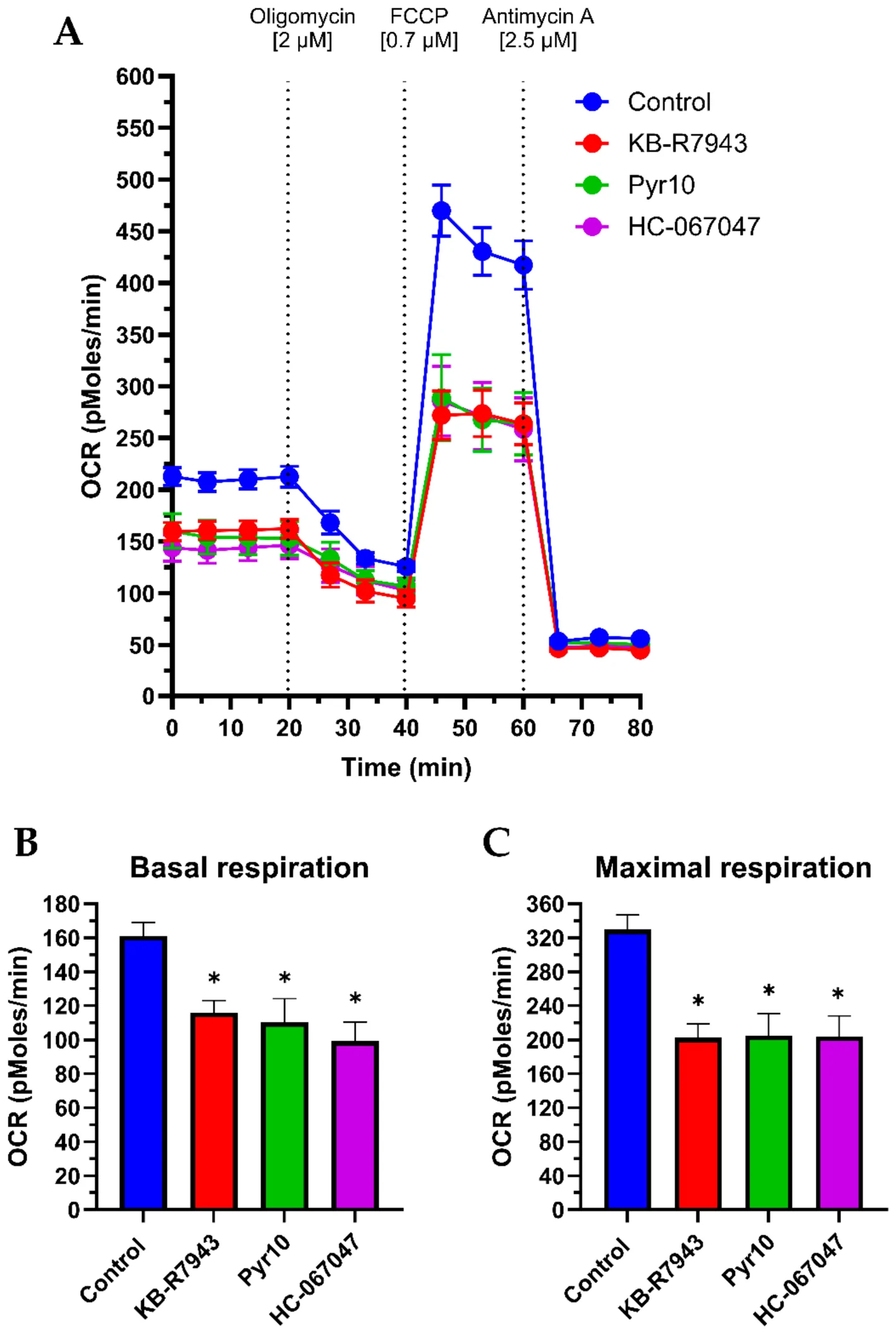
NCX and TRP channel activity are required to sustain mitochondrial respiration in INS-1 β-cells. (**A**) Average Seahorse XF96 traces showing oxygen consumption rates (OCR) of INS-1 cells under control (blue), KB-R7943 (red), Pyr10 (green), and HC-067047 (purple) conditions. Dashed lines indicate injections of Oligomycin, FCCP, and Antimycin A. (**B**) Bar graphs show basal respiration and (**C**) maximal respiration levels. Data points represent mean ± SEM (n is greater than or equal to 6 independent experiments). Significant differences were assessed with one-way ANOVA with Bonferroni post hoc test (* *p* < 0.05 versus control).

**Figure 10:**
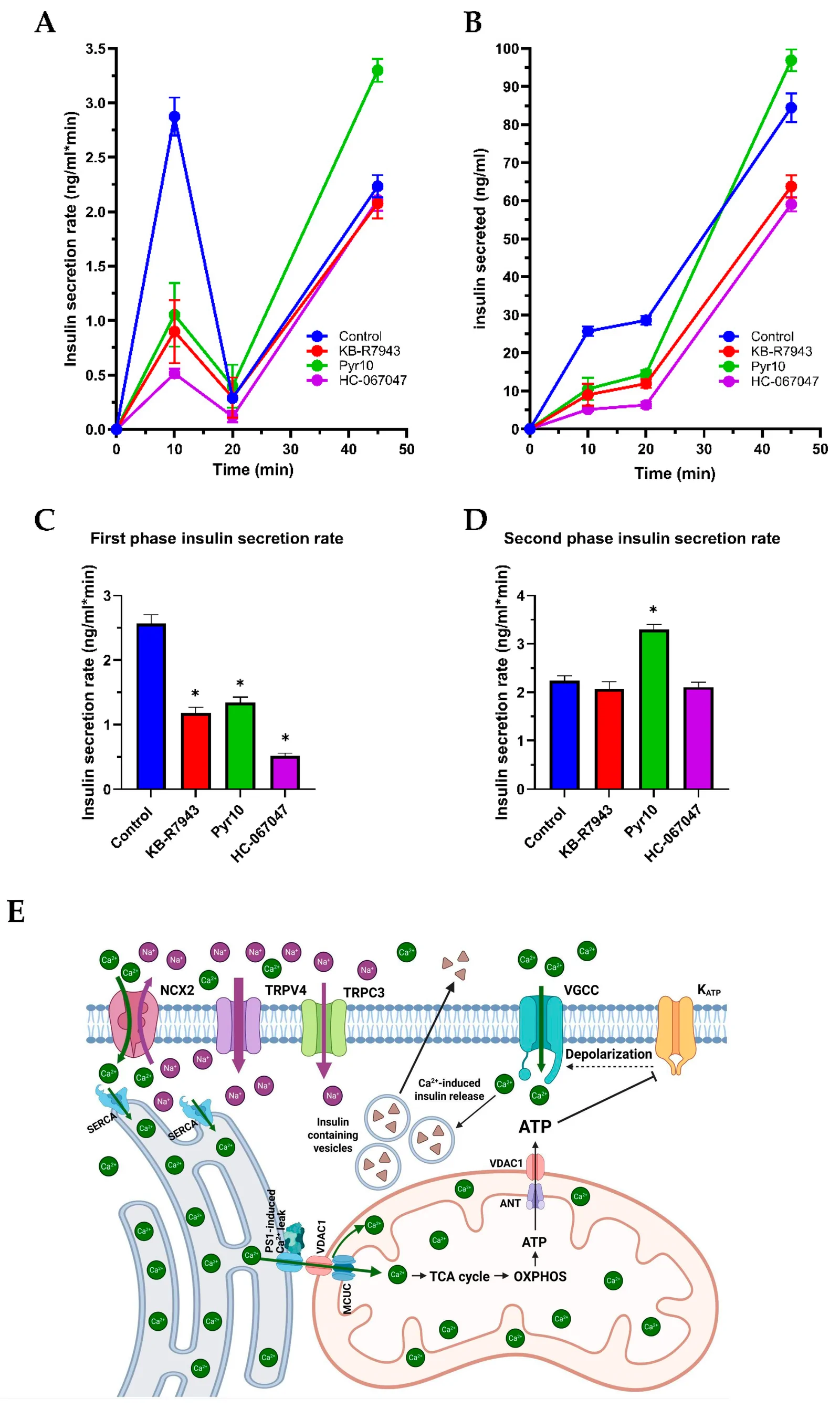
NCX and TRP channels regulate first-phase glucose-stimulated insulin secretion in INS-1 β-cells. (**A**) Time-dependent insulin secretion rate (nanograms per milliliter per minute) and (**B**) cumulative secreted insulin (nanograms per milliliter) under control (blue), KB-R7943 (red), Pyr10 (green), and HC-067047 (purple) conditions. Insulin secretion at time point “0” min was set to 0 ng/mL. (**C**) Bar graphs show the first-phase insulin secretion rate (10–20 min) and (**D**) second-phase insulin secretion rate (20–45 min). Data points represent mean ± SEM (n is greater than or equal to 4). Significant differences were assessed using one-way ANOVA with Bonferroni post hoc test (* *p* < 0.05 versus control). (**E**) Schematic model of the TRPs–NCX2 relay maintaining beta-cell glucose sensitivity and ER-mitochondria bioenergetics.

## Data Availability

The original data presented in the study will be openly available. Access is provided upon reasonable request from the corresponding author.

## Supplementary Materials

The following supporting information can be downloaded at: https://www.mdpi.com/article/10.3390/cells15191766/s1, Figure S1: ORAI1, but not NCX2, is required for store-operated Ca^2+^ entry in EndoC-βH5 cells; Figure S2: Measurement of STIM1 oligomerization in INS1 cells using FRET; Figure S3: STIM1 and STIM2 are dispensable for basal ER Ca^2+^ content.

## Abbreviations

The following abbreviations are used in this manuscript:

PS1	presenilin-1
GSK3β	glycogen synthase kinase 3 beta
ER	Endoplasmic reticulum
STIM	stromal interaction molecule
ORAI	Ion channel responsible for store-operated Ca^2+^ entry
INS-1	immortalized rat insulinoma model cell line
TRP	transient receptor potential channels
NCX	plasma membrane sodium-calcium exchanger
SERCA	sarcoplasmic/endoplasmic reticulum calcium ATPase
TCA	tricarboxylic acid cycle
Cch	carbachol
BHQ	2,5-di-tert-butylhydroquinone
