# Supplementary material for: TRPs—NCX2 Axis Jointly Preserves Pancreatic Beta Cell Susceptibility/Sensitivity to Elevated Glucose
Source: Cells. 2026 Sep 28;15(19):1766. doi: 10.3390/cells15191766 (PMC13663930; doi:10.3390/cells15191766)
Supplement: Supplementary file 1 [file cells-15-01766-s001.zip › cells-4533295-supplementary.pdf]

Supplementary Figures

TRPs – NCX2 axis jointly preserves pancreatic beta cell susceptibility/sensitivity to elevated glucose

Adlet Sagintayev[1], Furkan E Oflaz[1], Andra Kanlinuro[1], Savina van Amsterdam[1], Benjamin Gottschalk[1], Rene Rost[1], Zhanat Koshenov[1], Roland Malli[2,3] & Wolfgang F Graier[1,3,*]

[1]Gottfried Schatz Research Center: Molecular Biology and Biochemistry, Medical University of Graz, Graz, Austria

[2]Core Facility Bioimaging, ZMF, Medical University of Graz, Graz, Austria

[3]BioTechMed-Graz, Graz, Austria

*Correspondence: wolfgang.graier@medunigraz.at; +43 316 385 71963

Supplementary Figure S1

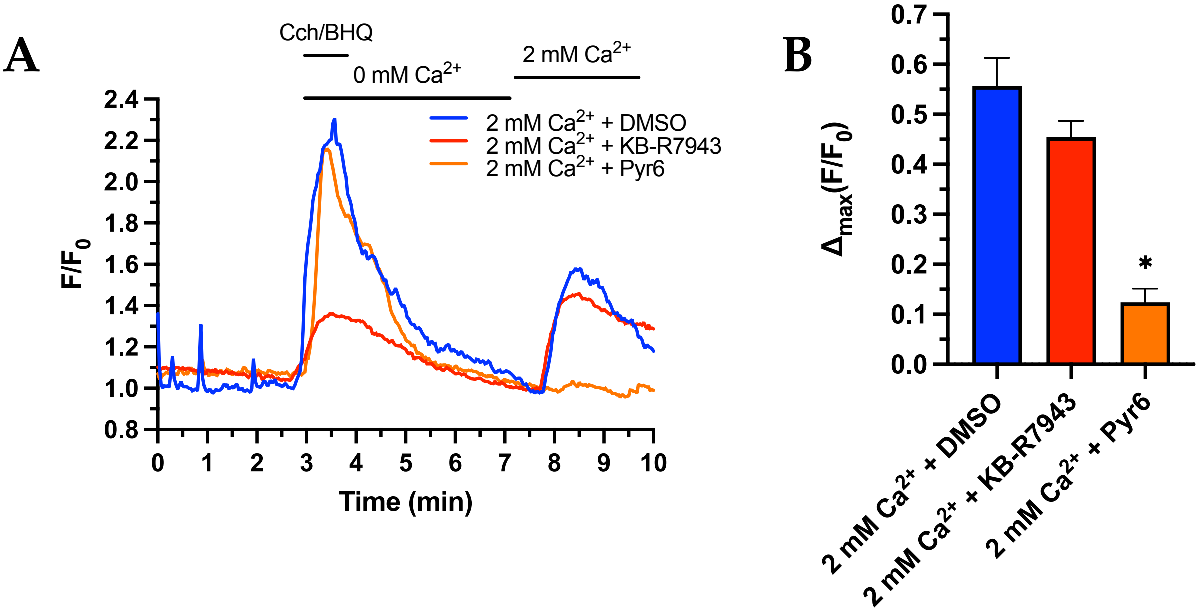

Supplementary Figure S1. ORAI1, but not NCX2, is required for store-operated Ca^{2+} entry in EndoC-βH5 cells (A) Representative Fluo-4 AM traces showing cytosolic Ca^{2+} ($[Ca^{2+}]_{cyto}$, F/F0) over time in EndoC-βH5 cells preincubated for 40 minutes in the respective buffer (2 mM Ca^{2+} + DMSO, blue; 2 mM Ca^{2+} + KB-R7943, red; 2 mM Ca^{2+} + Pyr6, orange). Cells were stimulated with 100 µM carbachol (Cch) and 15 µM BHQ in 0 mM Ca^{2+} solution to deplete ER Ca^{2+} stores. Extracellular Ca^{2+} was then restored to trigger store-operated Ca^{2+} entry. **(B)** Bar graph shows the peak amplitude of the cytosolic Ca^{2+} rise upon Ca^{2+} readdition (Δmax, F/F0) for each condition. Data represent mean +/- SEM (n for 2 mM Ca^{2+} + DMSO = 42/3; 2 mM Ca^{2+} + KB-R7943 = 33/3; 2 mM Ca^{2+} + Pyr6 = 43/3). Significant differences were assessed using one-way ANOVA with Bonferroni post hoc test (*$p < 0.05$ versus 2 mM Ca^{2+} + DMSO control).

Supplementary Figure S2

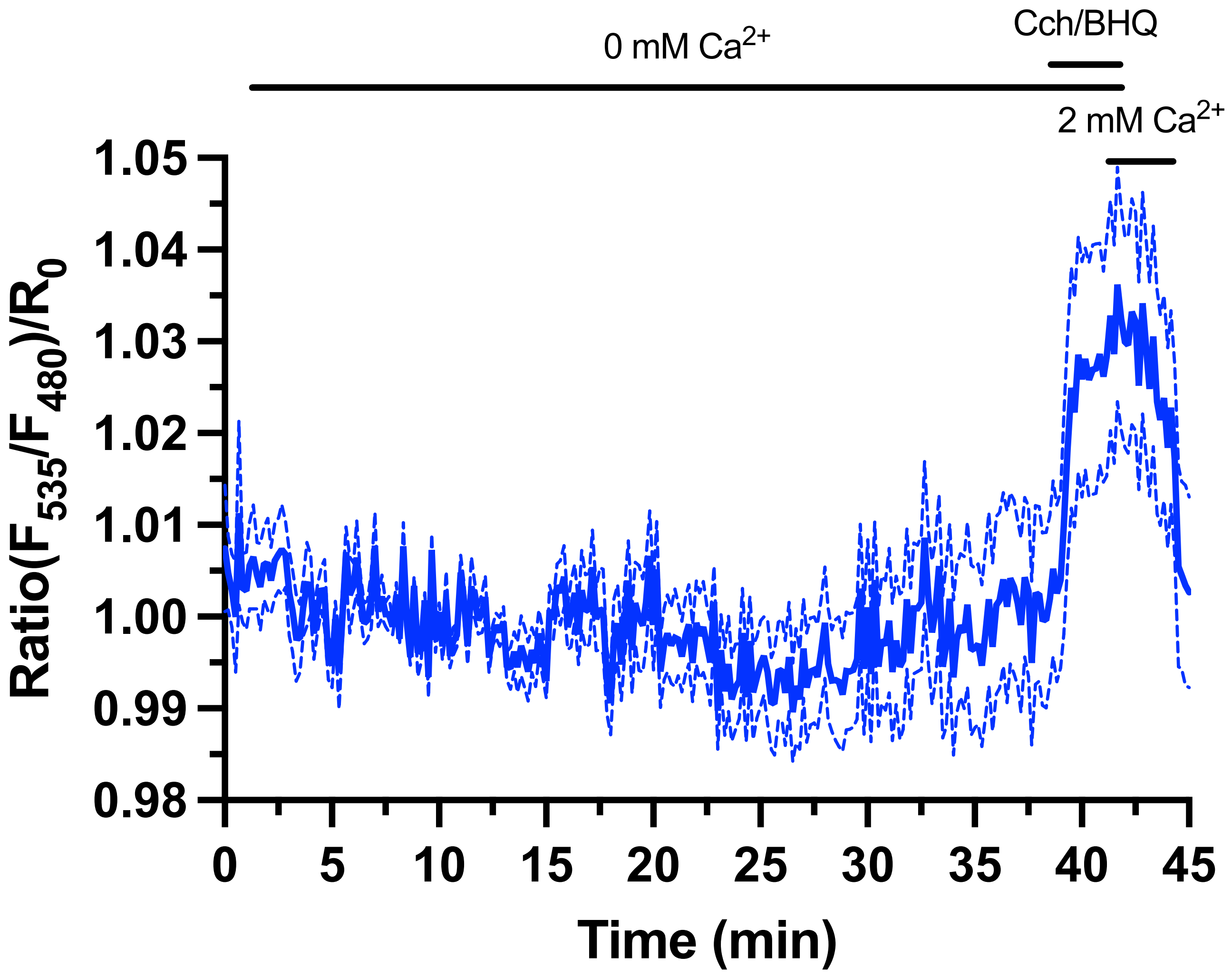

Supplementary Figure S2. Measurement of STIM1 oligomerization in INS1 cells using FRET. Time course of FRET ratio changes in INS1 cells co-expressing CFP-STIM1 and YFP-STIM1 over a 45-minute recording period. Cells were initially bathed in standard buffer containing 2 mM Ca^{2+} for 2 minutes, followed by removal of extracellular calcium with 0 mM Ca^{2+} buffer containing 0.1 mM EGTA for 35 minutes (minutes 2–37). To induce endoplasmic reticulum (ER) calcium store depletion, carbachol (100 µM) and 2,5-di-t-butylhydroquinone (15 µM) (Cch/ BHQ) were applied in 0 mM Ca^{2+} buffer for 5 minutes (minutes 37–42), followed by re-addition of 2 mM Ca^{2+} buffer for the final 3 minutes (minutes 42–45). Data represent the normalized emission ratio (Ratio(F_{535}/F_{480})/R_0) expressed as the mean +/- SEM (solid line and dashed error bands, respectively) from n = 8 cells across 3 wells from 1 experiment day.

Supplementary Figure S3

A

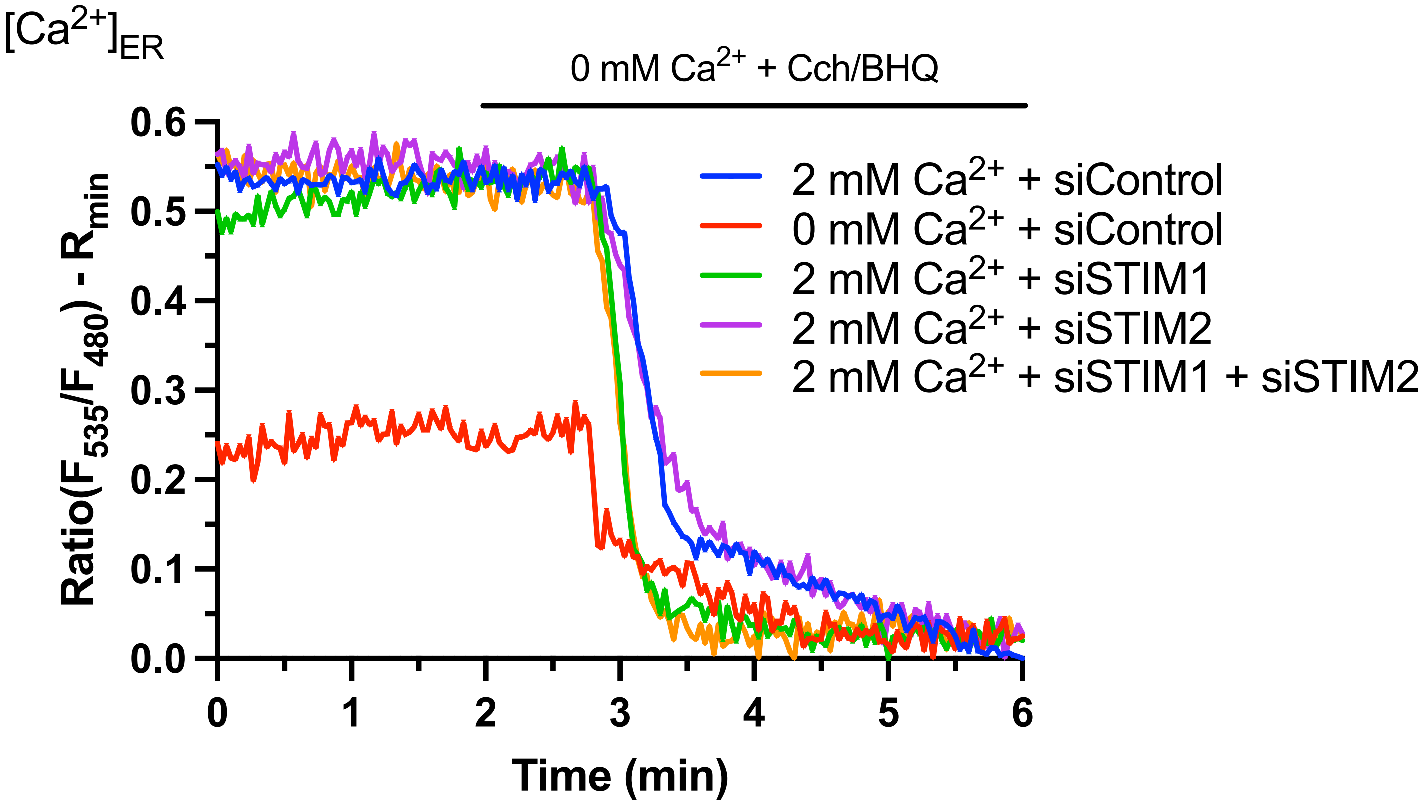

B

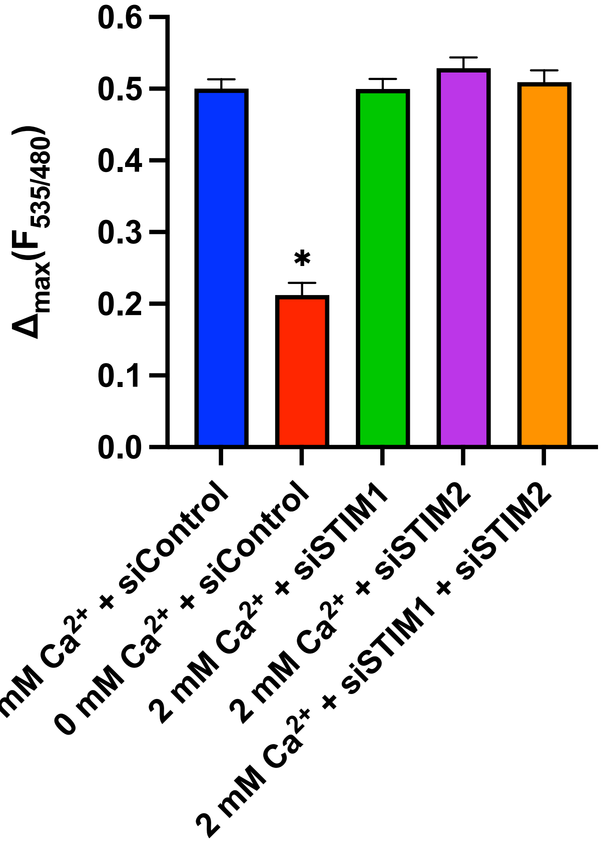

Supplementary Figure S3. STIM1 and STIM2 are dispensable for basal ER Ca^{2+} content
(A) Representative D1ER FRET traces showing ER Ca^{2+} content (Ratio(F535/F480) − Rmin, where Rmin is the minimum ratio obtained after maximal store depletion) over time in INS-1 832/13 cells transfected with control siRNA (siControl) or siRNA against STIM1, STIM2, or both, and preincubated for 40 minutes in 2 mM Ca^{2+} (siControl, blue; siSTIM1, green; siSTIM2, purple; siSTIM1 + siSTIM2, orange) or 0 mM Ca^{2+} (siControl, red). At 2 minutes, cells were stimulated with 100 µM carbachol (Cch) and 15 µM BHQ in 0 mM Ca^{2+} solution to fully deplete ER Ca^{2+} stores. **(B)** Bar graph shows the basal ER Ca^{2+} content (Δmax, F535/F480) prior to store depletion for each condition. Data represent mean +/- SEM (n for 2 mM Ca^{2+} + siControl = 46/6; 0 mM Ca^{2+} + siControl = 32/6; 2 mM Ca^{2+} + siSTIM1 = 36/6; 2 mM Ca^{2+} + siSTIM2 = 40/6; 2 mM Ca^{2+} + siSTIM1 + siSTIM2 = 33/6). Significant differences were assessed using one-way ANOVA with Bonferroni post hoc test (*$p < 0.05$ versus 2 mM Ca^{2+} + siControl).
